# 5000 years of dietary variations of prehistoric farmers in the Great Hungarian Plain

**DOI:** 10.1371/journal.pone.0197214

**Published:** 2018-05-10

**Authors:** Beatriz Gamarra, Rachel Howcroft, Ashley McCall, János Dani, Zsigmond Hajdú, Emese Gyöngyvér Nagy, László D. Szabó, László Domboróczki, Ildikó Pap, Pál Raczky, Antónia Marcsik, Zsuzsanna K. Zoffmann, Tamás Hajdu, Robin N. M. Feeney, Ron Pinhasi

**Affiliations:** 1 School of Archaeology, University College Dublin, Dublin, Ireland; 2 Earth Institute, University College Dublin, Dublin, Ireland; 3 Conway Institute, University College Dublin, Dublin, Ireland; 4 Déri Museum, Debrecen, Hungary; 5 István Dobó Castle Museum, Eger, Hungary; 6 Department of Anthropology, Hungarian Natural History Museum, Budapest, Hungary; 7 Insitute of Archaeological Sciences, Faculty of Humanities, Eötvös Loránd University, Budapest, Hungary; 8 University of Szeged, Szeged, Hungary; 9 Hungarian National Museum, Budapest, Hungary; 10 Department of Biological Anthropology, Institute of Biology, EötvösLoránd University, Budapest, Hungary; 11 School of Medicine, University College Dublin, Dublin, Ireland; Museo delle Civiltà, ITALY

## Abstract

The development of farming was a catalyst for the evolution of the human diet from the varied subsistence practices of hunter-gatherers to the more globalised food economy we depend upon today. Although there has been considerable research into the dietary changes associated with the initial spread of farming, less attention has been given to how dietary choices continued to develop during subsequent millennia. A paleogenomic time transect for 5 millennia of human occupation in the Great Hungarian Plain spanning from the advent of the Neolithic to the Iron Age, showed major genomic turnovers. Here we assess where these genetic turnovers are associated with corresponding dietary shifts, by examining the carbon and nitrogen stable isotope ratios of 52 individuals. Results provide evidence that early Neolithic individuals, which were genetically characterised as Mesolithic hunter-gatherers, relied on wild resources to a greater extent than those whose genomic attributes were of typical Neolithic European farmers. Other Neolithic individuals and those from the Copper Age to Bronze Age periods relied mostly on terrestrial C_3_ plant resources. We also report a carbon isotopic ratio typical of C_4_ plants, which may indicate millet consumption in the Late Bronze Age, despite suggestions of the crop’s earlier arrival in Europe during the Neolithic.

## Introduction

**Fig 1:**
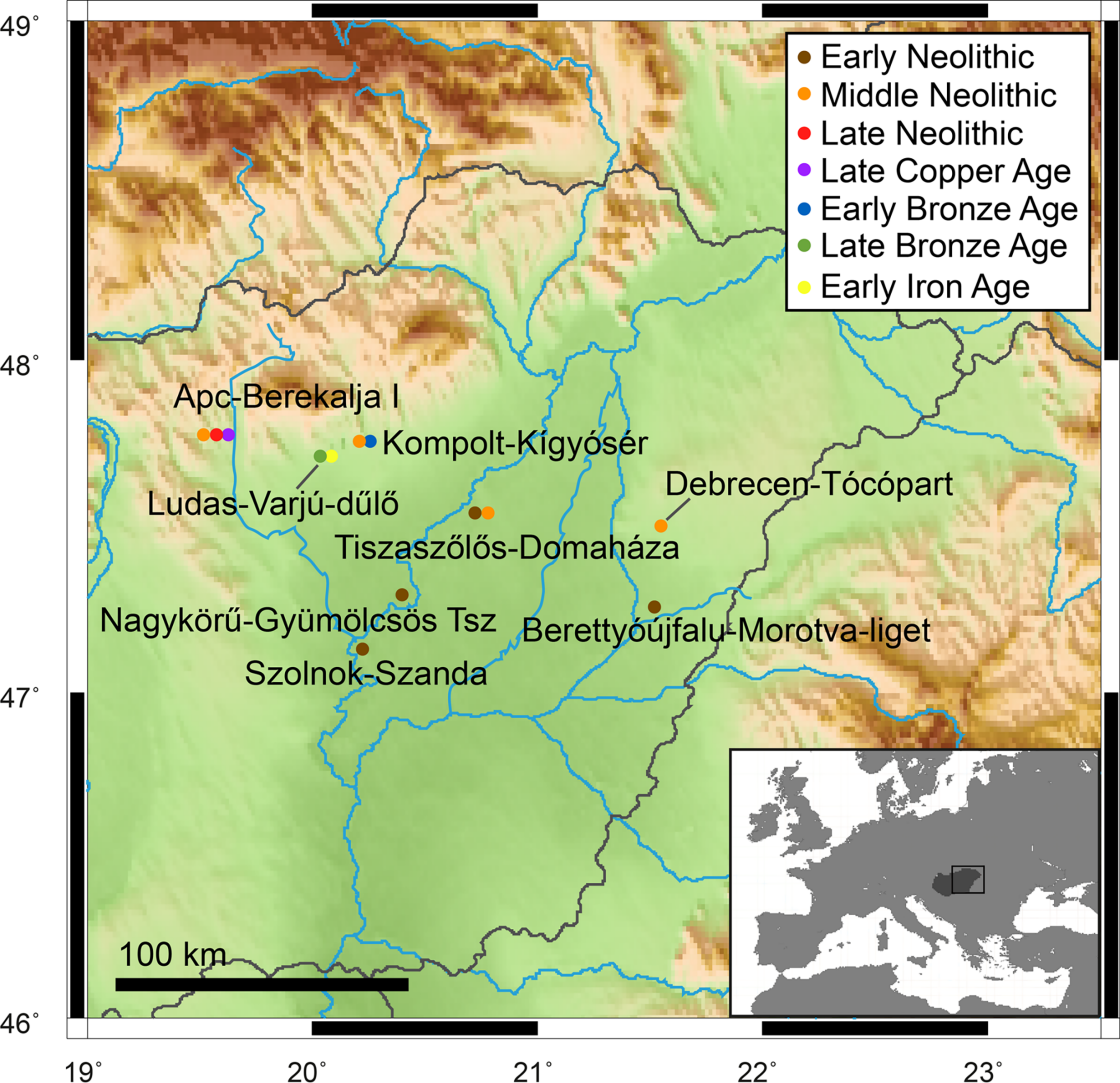
Map showing the location of sites analysed in the study. Generic Mapping Tools 4.5.13 [11] and the topographic ETOPO data set [12] was used to create this map.

The transition to agriculture was one of the most significant events in human history, driving major biological and cultural changes globally. A key element in most agricultural transitions is a sedentary lifestyle, which is typically accompanied by changes in subsistence from hunting and gathering to farming [1]. The nature, duration, and timing of the onset of agriculture varied across Europe (see [2–4]) as did the technological, cultural, and biological changes associated with this transition [5]. Agriculture spread into Europe from northwestern Anatolia and the Near East during the first half of the 7^th^ millennium BC [3] and reached the Carpathian Basin ~6,000 BC [6]. The Great Hungarian Plain (GHP), also known as the Nagy-Alföld, is a lowland situated centrally in the Carpathian Basin, east of the Danube, and connected with the Mediterranean, the Pontic steppe and Central Europe [7,8] (Fig 1). The GHP was a key area involved in the spread and development of farming across Europe. This area was the meeting point of eastern and western European cultures, and as such was a major cultural and technological transitional region throughout prehistory [9,10].

Gamba et al. [13] analysed the genomes of nine Neolithic, one Copper Age, two Bronze and one Iron Age (5,800–830 cal BC) burial(s) spanning a 5,000-year temporal transect from the Early Neolithic to Early Iron Age. They evaluated the interface between genetic changes brought on by migrations and interactions during these key techno-cultural transitions in the GHP. Interestingly, although eight of the nine Neolithic and Copper Age individuals were genetically affiliated with modern-day Sardinians (a pattern which has been observed for numerous other European Neolithic farmers [14–17]), one of the Early Neolithic Körös individuals analysed has a genomic profile characteristic of an non-admixed European Mesolithic hunter-gatherer. This unexpected result suggests that this Early Neolithic individual may not have had shared the same subsistence practices as other early and later Neolithic individuals. Their study also showed genomic turnovers coinciding with the advent of the Bronze and Iron Ages, which contrasted with ~2,800-years of genetic continuity during the preceding Neolithic and Copper Age periods.

### Archaeological context and subsistence practice in the Great Hungarian Plain

Agriculture arrived in the GHP with the appearance of the Early Neolithic Körös culture [2,18] (see Table 1 for more details). This was the northernmost expression of the Balkan Early Neolithic complex (Starčevo-Körös-Criş cultures) that had spread farming throughout south-east Europe from its origins in the Near East [2]. Körös subsistence strategies were predominantly based on grain cultivation (wheat, barley, einkorn and legumes) and animal husbandry [2,19]. Most of the Körös faunal assemblages suggest that these early farmers had a characteristically southeastern European subsistence that was heavily reliant on sheep and goat husbandry, followed by cattle contribution (although in some Körös sites, the number of cattle bones was higher than ovicaprids [20]), and a very limited use of pigs and wild resources [2,21]. The Neolithic Linearbandkeramik culture (LBK) appeared in Transdanubia (West Hungary) and spread across the loess plains of Central Europe westward to the Paris Basin and eastward to the Ukraine, and eventually being responsible for establishing farming across much of the northern regions of Europe [22,23]. The Alföld Linear Pottery culture (ALP) was a LBK variant culture in the GHP. The subsistence practice was characterized by a major reliance on cattle [2,21,23], as well as cereals (mainly: emmer, barley, einkorn), legumes (pea and lentils), and flax cultivation [2,23–25]. The ALP culture was succeeded by the Late Neolithic Tisza–Herpály–Csőszhalom complex [26]. The range of subsistence practices was similar to the previous ALP, with reliance on grain cultivation and an emphasis on domesticated cattle at the expense of ovicaprids (sheep and goats). Additionally, it included an increase of wild resources consumption [21].

**Table 1:** Summary of the prehistoric time periods and their associated cultures and subsistence practices in the Great Hungarian Plain.

Time Period	Date Range	Associated Cultures	Subsistence practices
Early Neolithic	6,500–5,500 BC	Körös	Grain cultivation (wheat, barley, einkorn) and animal husbandry (predominantly: sheep/goat)
Middle Neolithic	5,500–5,000 BC	Linearbandkeramik (LBK), Alföld Linear Pottery (ALP)	Grain cultivation (wheat, barley, einkorn) and animal husbandry (major reliance on cattle)
Late Neolithic	5,000–4,500 BC	Tisza, Herpály, Csőszhalom	Grain cultivation (wheat, barley, einkorn); animal husbandry with emphasis on domesticated cattle
Early Copper Age	4,500–4,000 BC	Tiszapolgar	Focus on animal husbandry (mainly cattle)
Middle Copper Age	4,000–3,500 BC	Bodrogkeresztúr	Focus on animal husbandry (mainly cattle)
Late Copper Age	3,500–2,700 BC	Baden	Focus on animal husbandry (mainly cattle)
Early/Middle Bronze Age	2,700 –,1400 BC	Nagyrév, Hatvan, Ottomány	Intensive crop cultivation and animal husbandry
Late Bronze Age	1,400–900 BC	Tumulus, Urnfield, Kyjatice	Intensive crop cultivation; millet as staple crop
Early/Middle Iron Age	900–450 BC	Mezőcsát	Pastoral nomadism, semi-nomadic or transhuman pastoralist. Stock breeding of gregarious animals (cattle, sheep or horses)

The transition from the Late Neolithic to the Copper Age in the GHP was characterised by an overall cultural continuity, but with some changes in settlement patterns [27,28] and subsistence, namely from a reliance on agricultural products to a focus on animal husbandry (mainly cattle). This shift became predominant especially during the Middle Copper Age [27,29].

Bronze metallurgy arrived to the Carpathian Basin in the middle of the 3^rd^ millennium BC, as a result of trade links with the northern Pontic and Balkan Peninsula [30,31]. During the Bronze Age, several new regional cultures appeared on the GHP in association with local technological developments, the emergence of extensive trade networks for metallurgy, and the arrival of various groups from the steppe [30,32–35]. The subsistence practices of the Early and Middle Bronze Age tell cultures (named for the new type of settlements tells or nucleated villages; e.g. Nagyrév, Hatvan, Ottomány and Perjámos in eastern Hungary) were characterised by intensive crop cultivation and animal husbandry [32,34,36]. This also includes domestic horses that arrived from steppe cultures, and the exploitation of freshwater resources [30,32]. The Late Bronze Age was a period of major social and economic change associated with pan-regional connections with cultures from the western part of Central Europe (e.g. Tumulus, Urnfield) [35–37]. The earliest archaeobotanical evidence of broomcorn millet is reported in China, dating 8,000 BC [38]. It was used as a staple crop in Neolithic northern China by 6,000 BC [39] and spread westward to Central Asia and Eastern Europe over time [40,41]. While some evidence suggests millet consumption by Early Neolithic individuals in the GHP [42,43], it was not until the middle of the 2^nd^ millennium BC that a shift was documented in cultivation preferences and millet was cultivated as a staple crop in the Carpathian Basin, as well as in continental Europe [36,44,45].

Iron metallurgy arrived in Central Europe during the first millennium BC [46]. During the Early and Middle Iron Ages, pre-Scythian (also referred as Mezőcsát communities) and Scythian cultures from the Eastern Steppe inhabited the GHP and the adjacent northern mountainous region [47]. These populations practiced a form of nomadic stockbreeding [47,48], leaving limited traces of material culture behind in the archaeological record. Mezőcsát groups who lived predominantly in the northern part of the GHP are suggested by some to be descendants of these nomadic groups [47], while others argue local continuity from the Late Bronze Age to the Middle Iron Ages, and the adoption of a pastoralist lifestyle as a result of contacts with eastern populations [48].

### Carbon and nitrogen stable isotope ratios as paleodietary indicators

The use of carbon and nitrogen stable isotope ratios to study paleodiet is based on the principle that the isotope values of food consumed by animals and humans are reflected in the individual’s tissues (see [49–51]). Moreover, as bone is constantly being turned over by remodelling, the stable isotope ratios of bone collagen are indicative of the average diet over a period of time prior to death that may extend many years or even decades in adult individuals [52]. In brief, carbon stable isotope ratios (δ^13^C; see S2 Appendix) are primarily used to distinguish between the consumption of C_4_ and C_3_ photosynthesizing plants, and the animals raised on them [53]. They also allow differentiation between the consumption of terrestrial and marine foods [54,55]. In terrestrial ecosystems, the range of δ^13^C in C_3_ plants, which include most trees, shrubs, temperate grasses, and domesticated cereals such as wheat, barley, oats, and rice, varies widely from −24‰ to −36‰ (mean −26.5‰) depending on environmental factors [56,57]. C_4_ plants are typically arid adapted species and include some domesticated plants like maize, sorghum, and millet. C_4_ plants tend to have less variable and higher δ^13^C values (mean −12.5‰) than C_3_ plants, and these values create a bimodal distribution making them distinguishable from each other. Although variation in δ^13^C values exists, freshwater fish consumption results in lower δ^13^C values than in terrestrial C_3_ environments, with a range of −23‰ to −21‰ δ^13^C values in Europe [58–60].

Nitrogen stable isotope values (δ^15^N) in tissues increase with trophic level, with a stepwise increase of 3–5‰ between diet and consumers. This trophic level increase is used to analyse the relative consumption of plant versus animal proteins in the diet [61,62]. Aquatic resources, from both marine and freshwater ecosystems, can be markedly ^15^N-enriched compared to terrestrial foods, due to longer and more complex food webs [58,63]. It is of relevance to note that various environmental factors including water stress (e.g. [64,65]), soil conditions (e.g. [66,67]), climate (e.g. [68,69]), health of the individual [70], or breastfeeding practices [71] have also shown to influence nitrogen isotope ratios. The use of animal manure additionally increases δ^15^N values in soils resulting in higher nitrogen isotope ratios in the tissues of animals and humans consuming fertilised crops [72–74].

Previous stable isotope research in Hungary has focused on Neolithic and Copper Age populations [22,25,42,75,76]. These studies indicate that during the Late Neolithic and Copper Age on the GHP, animal protein featured more prominently in peoples’ diet than in previous Early and Middle Neolithic periods [25,42,76]. Additionally, terrestrial C_3_ plants made most of the contribution to individual’s diets, except for some sites where some individuals’ δ^13^C values suggest an increase in C_4_ plant consumption (e.g. millet) [42]. Likewise, freshwater resources probably did not contribute significantly to Neolithic and Copper Age diets [25]. However, no palaeodietary isotopic studies included samples from Bronze and Iron Ages from the eastern GHP and have analysed dietary changes associated with the arrival of new material culture and migration events.

This study includes isotopic data from a 5,000 years transect of the GHP, including Bronze and Iron Age periods, and analyses dietary changes along prehistoric time in the GHP. Specifically, the aim of this study is to investigate if the genetic changes and cultural transitions occurred in the GHP from Early Neolithic to Iron Age were associated with dietary shifts. The analysis focuses on carbon and nitrogen stable isotope ratios of bone collagen of 13 individuals reported in [13], together with 39 additional individuals from the same sites. We found that changes in dietary patterns were not always accompanied by cultural and genetic shifts. We also report higher consumption of C_4_ plant resources in the Late Bronze and Iron Age periods.

## Material and methods

Carbon and nitrogen stable isotope analysis was carried out on collagen from bone samples of 52 human individuals from both sexes and various ages, along with 17 faunal bone samples representing 6 species from three settlements (Tables 2 and 3; see details in S1 and S2 Tables). All necessary permits were obtained for the described study, which complied with all relevant regulations. Only data from adults and subadults >4 years old were used for statistical purposes in order to avoid individuals that might have still been breastfeeding or in the weaning process by the time of death [71]. The samples span from the early Neolithic Körös culture to the Early Iron Age Mezőcsát culture. We combined previous isotopic data for humans and fauna from the same region with the new data from this study in order to increase sample sizes and to analyse diachronic patterns of dietary change (detailed in Tables 2 and 3). Published data without associated ages of individuals were not used. The variability of isotopic data from sites of the same time period is likely to be due to localvariations in the isotope composition of the food web, rather than a difference in subsistence practice as shown by [25]. Nevertheless, human samples from the Körös Early Neolithic site of Tiszaszőlős-Domaháza were compared separately from the rest of Early Neolithic individuals to test if their diet was significantly different, as their hunter-gatherer genetic affinity suggests.

**Table 2:** Site information of human samples used in this study from Mesolithic to Early Iron Age.

Site	Period	Culture	N	References
Szolnok-Szanda	EN	Körös	3	This study
Berettyóújfalu-Morotva-liget	EN	Körös	2	This study
Tiszaszőlős-Domahàza	EN; MN	Körös; Alföld Linear Pottery	4	This study
Nagykörű-Gyümölcsös TSZ	EN	Körös	1	This study
Ludas-Varjú-Dűlő	LBA; EIA	Kyjatice; Mezőcsát	18	This study
Debrecen-Tócópart, Erdőalja	MN	Alföld Linear Pottery	8	This study
Kompolt-Kígyósér, Kistér	MN, EBA	Alföld Linear Pottery; Makó or Hatvan	6	This study
Apc-Berekalja I	MN; LN; LCA; EBA	LBK; Lengyel; Baden; Makó or Hatvan	8	This study
Apc-Berekalja II	MN	LBK	1	This study
Maroslele-Pana	MES; EN	Körös	5	[76]
Deszk	EN	Körös	2	[76]
Szarvas 23	EN	Körös	1	[76]
Endrőd-Varyai-tanya	EN	Körös	1	[76]
Mezőkövesd-Mocsolyás	MN	Alföld Linear Pottery	4	[22]
Füzesabony-Gubakút	MN	Alföld Linear Pottery	10	[22]
Polgár-Ferenczi-hát	MN	Alföld Linear Pottery	42	[22]
Hódmezővásárhely-Gorzsa	LN	Tisza; proto-Tiszapolgár	10	[25]
Kisköre-Gát	LN	Tisza	10	[25]
Polgár-Csőszhalom*	LN	Csőszhalom	9	[25]
Vésztő-Mágor and Vésztő-Bikeri	LN; ECA	Tisza; Tiszapolgár	22	[25]
Tiszapolgar-Basatanya	ECA; MCA	Tiszapolgár; Bodrogkeresztúr	20	[25]
Hajdúböszörmény-Ficsori-tó	ECA	Tiszapolgár	10	[25]
Magyarhomoróg	MCA	Bodrogkeresztúr	10	[25]

N = number of samples; MES = Mesolithic; EN = Early Neolithic; MN = Middle Neolithic; LN = Late Neolithic; ECA = Early Copper Age; MCA = Middle Copper Age; LCA = Late Copper Age; EBA = Early Bronze Age; LBA = Late Bronze Age; EIA = Early Iron Age.

*Only one individual from this site was not included as it has an anomalous δ^15^N ‰ value [25].

**Table 3:** Site information of faunal samples used in this study from Early Neolithic to Early Copper Age.

Species	N	Period	Sites	References
Cattle	51	Early Neolithic	Ecsegfalva; Berettyóújfalu-Morotva-liget; Szolnok-Szanda	[22,25,42], this study
		Middle Neolithic	Füzesabony-Gubakút; Polgár-Ferenczi-hát; Polgár-Piócási-dűlő; Debrecen-Tócópart, Erdőalja	
		Late Copper Age	Abony36; Vésztő-Bikeri	
Sheep/goat	39	Early Neolithic	Ecsegfalva; Endrőd 119; Berettyóújfalu-Morotva-liget; Szolnok-Szanda	[22,25,42,76], this study
		Middle Neolithic	Füzesabony-Gubakút; Polgár-Ferenczi-hát; Polgár-Piócási-dűlő	
		Late Copper Age	Abony3;, Vésztő-Bikeri	
Donkey	2	Middle Neolithic	Berettyóújfalu-Morotva-liget	This study
Auroch	5	Early Neolithic	Ecsegfalva; Berettyóújfalu-Morotva-liget	[25,42], this study
		Middle Neolithic	Polgár-Piócási-dűlő	
		Late Copper Age	Vésztő-Bikeri	
Red deer	6	Early Neolithic	Berettyóújfalu-Morotva-liget	[25], this study
		Middle Neolithic	Polgár-Piócási-dűlő	
		Late Copper Age	Abony36; Vésztő-Bikeri	
Pig	24	Early Neolithic	Berettyóújfalu-Morotva-liget; Szolnok-Szanda	[22,42], this study
		Middle Neolithic	Füzesabony-Gubakút; Polgár-Ferenczi-hát; Polgár-Piócási-dűlő; Debrecen-Tócópart, Erdőalja	
		Late Copper Age	Abony36; Vésztő-Bikeri	
Wild boar	6	Early Neolithic	Ecsegfalva	[25,42]
		Middle Neolithic	Polgár-Piócási-dűlő	
		Late Copper Age	Vésztő-Bikeri	
Fish	10	Early Neolithic	Ecsegfalva	[25,42]
		Middle Neolithic	Polgár-Piócási-dűlő	
		Late Copper Age	Abony36; Vésztő-Bikeri	

N = number of samples.

Collagen extraction followed a modified Longin method [77] (described in detail in S2 Appendix). Stable isotope analysis was carried out following the routine procedures of Light Stable Isotope Mass Spectrometry Laboratory of the Department of Geological Science at University of Florida (Gainesville, USA) and the University of Bradford Stable Light Isotope Laboratory (Bradford, UK) (described in S2 Appendix). The atomic C:N ratio range of 2.9−3.6 was used for quality control of the bone collagen, as it corresponds to well-preserved samples [78]. The distribution of isotopic values obtained was tested for normality using Shapiro-Wilk test, using *p*<0.05 at the statistical significance level (S3 Table). Statistical analyses (ANOVA and HSD Tukey post-hoc tests) were performed to assess differences between samples belonging to different periods. For those periods that do not follow a normal distribution, non-parametric Kruskal-Wallis and Mann Whitney U tests (for mean comparisons) were used. Statistical data were generated using SPSS version 24 and PAST version 3.16 [79]. All data generated or analysed during this study are included in this published article (and in Supporting Information files).

## Results

Bone collagen was obtained for 48 of the 52 human samples and for all faunal samples. Only one human sample did not have an atomic C:N ratio within the acceptable quality range of 2.9–3.6 [78] and its isotopic data were not considered for further statistical analyses and discussion. The information on faunal and human data obtained for this study are summarised in Table 4, and detailed in S1 and S2 Tables.

**Table 4:** Summary of results of the new isotopic data for the fauna (domesticates and wild species) and humans (per period) reported in this study.

	δ^13^C ‰					δ^15^N ‰				
Fauna	N	Min	Max	M	SD	N	Min	Max	M	SD
Domesticated	13	−21.2	−19.4	−20.4	0.5	13	5.7	9.5	7.0	1.3
Wild terrestrial	2	−21.8	−21	−21.4	0.5	2	6.1	7.1	6.6	0.7
**Humans**										
Early Neolithic	8	−22.6	−20.2	−21.0	1.0	8	8.8	13.1	11.1	1.4
Middle Neolithic	15	−20.5	−19.2	−19.9	0.4	15	9.2	12.2	10.6	0.8
Late Neolithic	2	−20.4	−20.0	−20.2	0.3	2	9.9	10.1	10.0	0.1
Late Copper Age	3	−20.5	−20.0	−20.3	0.3	3	10.1	10.4	10.3	0.2
Early Bronze Age	2	−19.9	−19.8	−19.9	0.1	2	10.7	11.5	11.1	0.6
Late Bronze Age	11	−19.0	−17.1	−17.9	0.6	11	10.2	11.6	10.9	0.5
Early Iron Age	3	−18.2	−14.4	−16.6	2.0	3	10.4	11.0	10.7	0.3

N = number of samples, Min = minimum, Max = maximum, M = mean, and SD = standard deviation.

### Isotope values of local fauna

Both domestic fauna (including ovicaprids, cattle and pig) and wild fauna (red deer, aurochs and wild boar) were used for statistical purposes, and are reported in S1 Table. The results are similar to those reported previously for other fauna from the same region and time periods. The stable isotope ratios of the local fauna analysed, with δ^13^C ranging from −21.8‰ to −19.4‰ and a mean value of −20.6‰ ±0.6‰ (1σ), are consistent with expected ranges for a terrestrial C_3_ environment [80]. The nitrogen isotopic values range from 5.7‰ to 9.5‰, with a mean value of 7.0‰ ±1.2‰ (1σ) (Table 4 and S1 Table).

**Fig 2:**
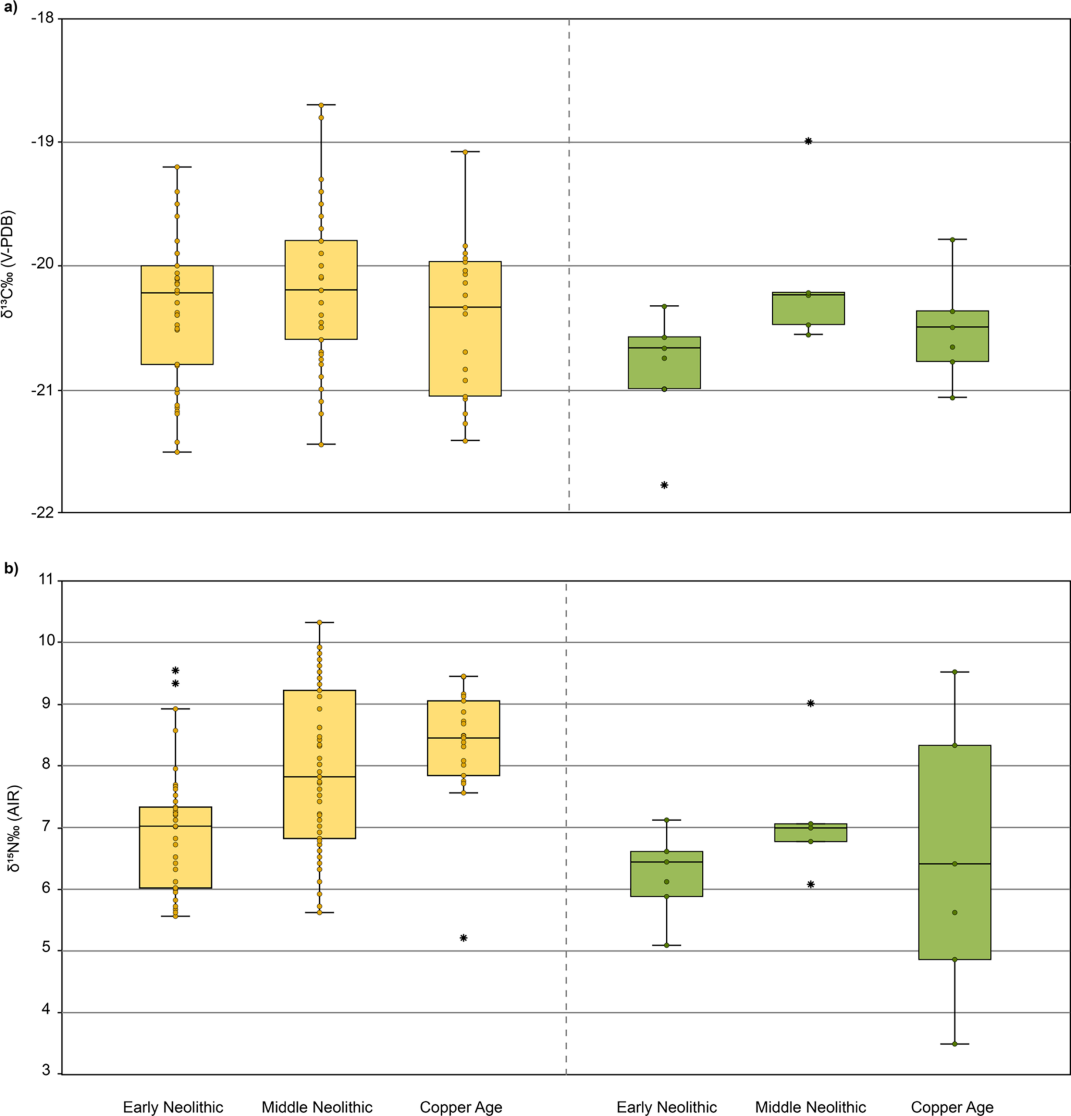
**Boxplot showing the (A) δ**^**13**^**C and (B) δ**^**15**^**N values of fauna samples from Early Neolithic to Copper Age**. Faunal data in this study were supplemented by published data of Early Neolithic [42,76], Middle Neolithic [22,25] and Copper Age [25] samples of the GHP. Domesticated fauna results on the left in yellow; wild fauna on the right in green. The dots within the boxes represent individual values of the samples; the horizontal line within the boxrepresents the median value; the vertical lines represent the range of data; and the asterisks are the possible outliers.

When comparing domesticates, no significant differences were found in carbon isotopic mean values over time (ANOVA: F = 2.631, *p* = 0.076) (Fig 2A). However, it appears that Middle Neolithic and Copper Age domesticated animals show higher δ^15^N values than those sampled from the Early Neolithic (Mann-Whitney U tests, *p*< 0.001) (Fig 2B). By contrast, wild fauna do not show significant isotopic changes over time (Kruskal-Wallis δ^13^C: H = 5.635, *p* = 0.060; ANOVA δ^15^N: F = 0.639, *p* = 0.542).

### Isotope values of the human samples

The human δ^13^C values reported here range from −22.6‰ to −14.4‰ (mean value of −19.4‰ ±1.5‰ (1σ)) and the δ^15^N values range between 8.8‰ and 13.1‰ (mean value 10.7‰ ±0.8‰ (1σ)) (Table 4 and S2 Table).

**Fig 3:**
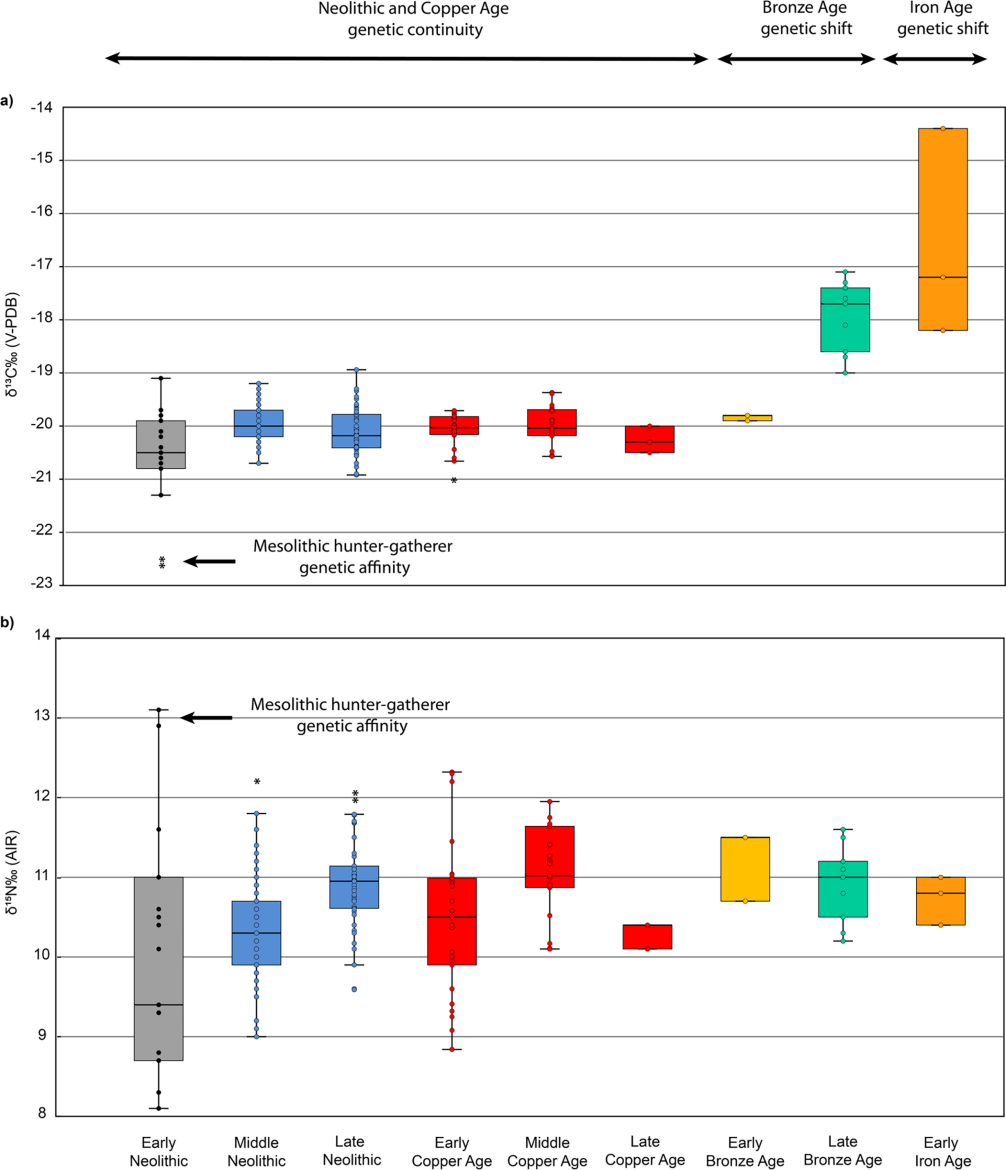
**Boxplot showing the (A) δ**^**13**^**C and (B) δ**^**15**^**N values of human samples from Early Neolithic to Early Iron Age**. Human isotopic values were combined with previous published data on the GHP from Early [76], Middle [22,25] and Late Neolithic [25], together with Early and Middle Copper Ages [25]. Genetic affinities are based on [13]. The dots within the boxes represent individual values of the samples; the horizontal line within the box represents the median value; the vertical lines represent the range of data.

**Fig 4:**
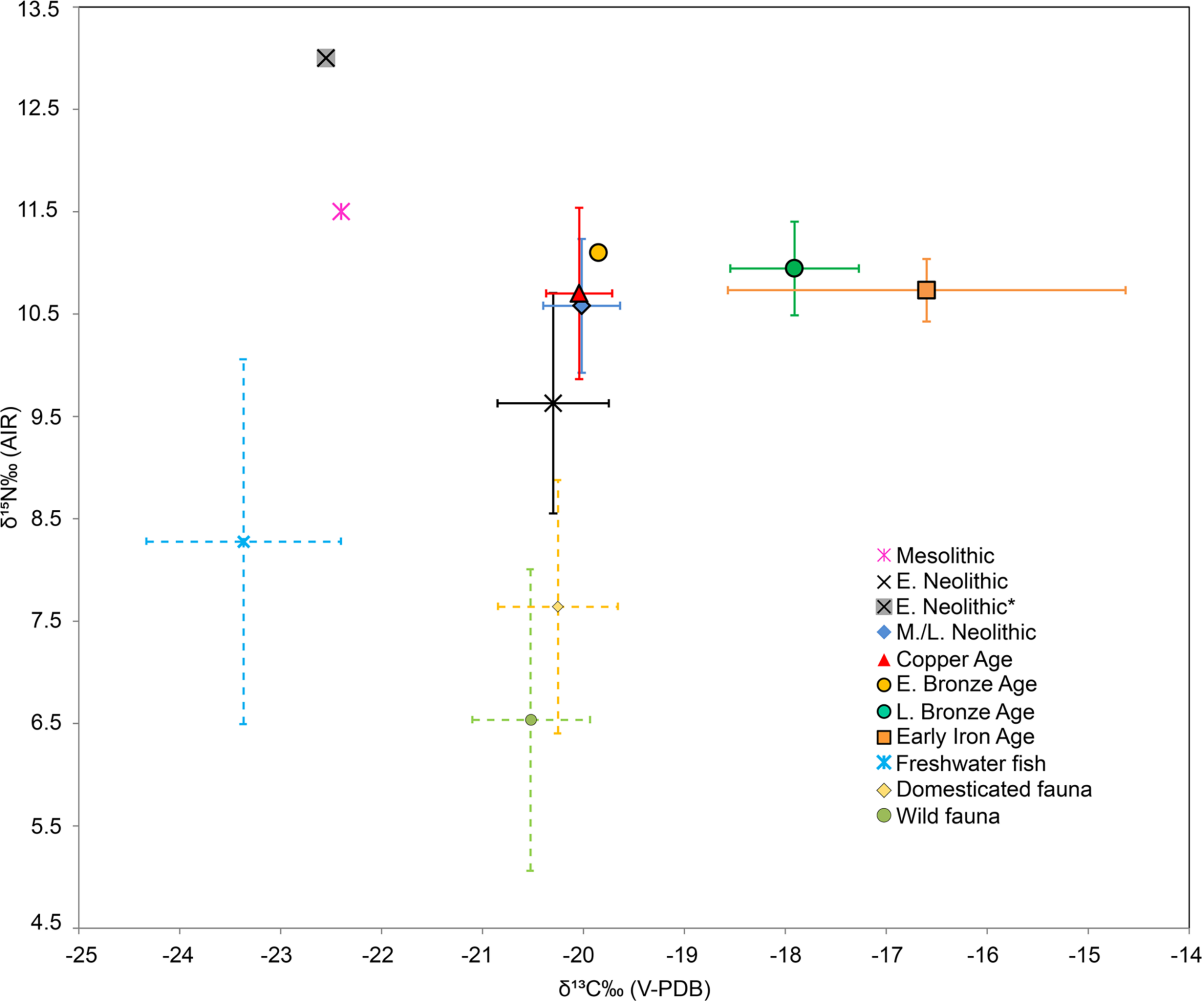
Stable carbon and nitrogen isotope data of human and faunal bone collagen from the GHP. Both human and fauna isotopic data are represented by mean isotopic values (standard deviation ±1σ indicated by bars). Domesticated and wild fauna belongs to Early/Middle Neolithic and Copper Age periods, and combined with published data [22,25,42,76]. Human results from this study were also combined with previous published data in the GHP [22,25,76]. E. Neolithic* represent mean values of Körös Tiszaszőlős-Domaháza site.

**Fig 5:**
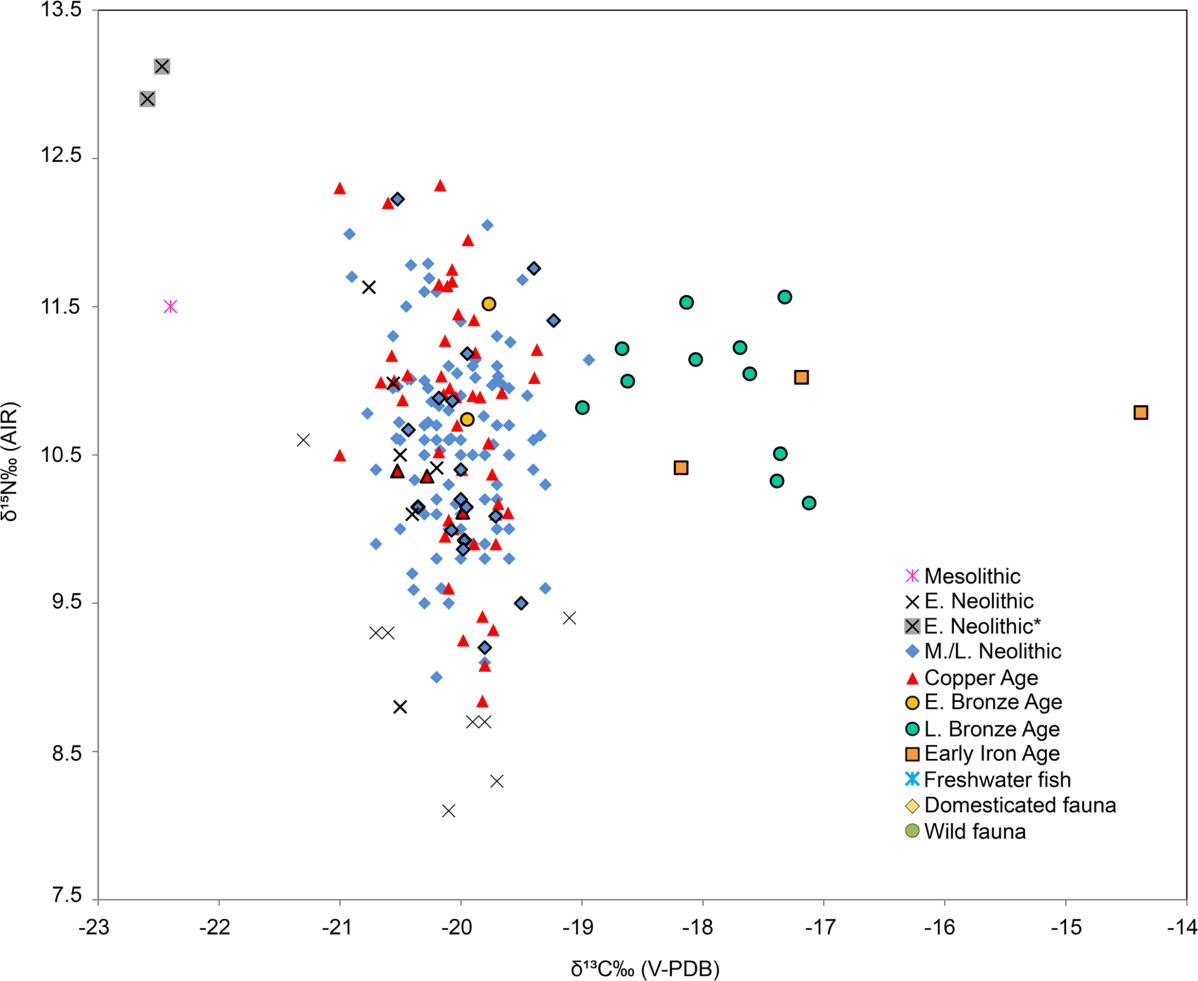
Stable carbon and nitrogen isotope data of human bone collagen from the GHP. Individual human results from this study (represented by highlighted symbols) were also combined with previously published data in the GHP [22,25,76]. E. Neolithic* represent mean values of the Körös Tiszaszőlős-Domaháza site.

When combining the current and published data, most of the individuals from Early Neolithic to Early Bronze Age have similar δ^13^C values consistent with a diet based on terrestrial C_3_ resources (Figs 3A and 4; S2 Table). However, Körös Early Neolithic individuals (n = 2) from the Tiszaszőlős-Domaháza site have significant lower δ^13^C values (*p*<0.05) than other Körös Early Neolithic and later period individuals (Table 5), similar to the Mesolithic individual from Maroslele-Pana [76] (Fig 5). By contrast, individuals from the Late Bronze Age and Iron Age have significantly (*p*<0.05) higher δ^13^C values than those reported for previous periods (Fig 3A, Table 5).

**Table 5:** Results of pairwise comparisons of δ^13^C ‰ values between human samples from different time periods (E = Early; M = Middle; L = Late).

	E. Neolithic*	E. Neolithic	M. Neolithic	L. Neolithic	*E*. *Copper Age*	M. Copper Age	L. Copper Age	E. Bronze Age	L. Bronze Age
E. Neolithic	**<0.001**								** **
M. Neolithic	**<0.001**	0.158							** **
L. Neolithic	**<0.001**	0.926	0.490						** **
*E*. *Copper Age*	***0*.*022***	*0*.*148*	*0*.*093*	*0*.*530*					*** ***
M. Copper Age	**<0.001**	0.485	1.000	0.966	*0*.*346*				** **
L. Copper Age	**<0.001**	1.000	0.953	1.000	*0*.*283*	0.979			** **
E. Bronze Age	**<0.001**	0.925	1.000	0.997	*0*.*265*	1.000	0.985		** **
L. Bronze Age	**<0.001**	**<0.001**	**<0.001**	**<0.001**	***<0*.*001***	**<0.001**	**<0.001**	**<0.001**	
E. Iron Age	**<0.001**	**<0.001**	**<0.001**	**<0.001**	***0*.*006***	**<0.001**	**<0.001**	**<0.001**	**<0.001**

All *p*-values correspond to HSD Tukey post-hoc comparisons, except for those from E. Copper Age samples (Mann-Whitney U test; in italics), as they do not follow a normal distribution (S3 Table). N = 199; E. Neolithic*: n = 2; E. Neolithic: n = 14; M. Neolithic: n = 71; L. Neolithic: n = 47; E. Copper Age: n = 26; M. Copper Age: n = 20; L. Copper Age: n = 3; E. Bronze Age: n = 2; L. Bronze Age: n = 11; E. Iron Age: n = 3. Values represent *p*-values (*p*<0.05 in bold).

*Early Neolithic samples from Tiszaszőlős-Domaháza site were treated separately for comparison.

When comparing samples from Early Neolithic to Middle Copper Age, individuals have a range of δ^15^N values suggesting varying amounts of animal protein intake, especially in the case of the Early Neolithic (Fig 3B). As indicated above, the Körös Tiszaszőlős-Domaháza individuals have the highest δ^15^N values, with significant differences than other Körös Early Neolithic and later period individuals, with the exception of Late Copper and Early Bronze Age individuals (Table 6). The mean δ^15^N values of Early Neolithic individuals (without including the Tiszaszőlős-Domaháza samples) have significantly lower values (*p*<0.05) than individuals from the Middle Neolithic to Middle Copper Ages (Table 6). At the same time, Late Neolithic samples, together with the Middle Copper Age ones, have significantly higher δ^15^N mean values (*p*<0.05) than the values for Middle Neolithic individuals (Fig 3B and Table 6). Individuals from the Late Copper Age have significantly lower δ^15^N values (*p*<0.05) than Late Neolithic and Middle Copper Ages samples (Table 6). Bronze and Iron Age δ^15^N values tend to be more restricted and less variable than previous periods. Late Bronze Age individuals have significantly higher δ^15^N values (*p*<0.05) than those of the Early Neolithic (Table 6).

**Table 6:** Results of pairwise comparisons of δ^15^N‰ values between human samples from different time periods (E = Early; M = Middle; L = Late).

	E. Neolithic*	E. Neolithic	M. Neolithic	L. Neolithic	E. Copper Age	M. Copper Age	*L*. *Copper Age*	E. Bronze Age	L. Bronze Age
E. Neolithic	**<0.001**								** **
M. Neolithic	**<0.001**	**0.014**							
L. Neolithic	**0.001**	**0.000**	**0.002**						
E. Copper Age	**<0.001**	**0.017**	1.000	0.178					
M. Copper Age	**0.011**	**<0.001**	**0.005**	0.990	0.090				* *
*L*. *Copper Age*	*0*.*200*	*0*.*432*	*0*.*918*	***0*.*032***	*0*.*866*	***0*.*026***			
E. Bronze Age	0.157	0.136	0.855	1.000	0.928	1.000	0.200		
L. Bronze Age	**0.008**	**<0.001**	0.230	1.000	0.570	1.000	0.060	1.000	
E. Iron Age	**0.018**	0.252	0.990	1.000	0.998	0.995	0.200	0.999	1.000

All *p*-values corresponds to HSD Tukey post-hoc comparisons, except for the ones from the L. Copper Age samples (Mann-Whitney U test; in italics), as they do not follow a normal distribution (S3 Table). N = 199; E. Neolithic: n = 2; E. Neolithic: n = 14; M. Neolithic: n = 71; L. Neolithic: n = 47; E. Copper Age: n = 26; M. Copper Age: n = 20; L. Copper Age: n = 3; E. Bronze Age: n = 2; L. Bronze Age: n = 11; E. Iron Age: n = 3. Values represent *p*-values (*p*<0.05 in bold).

*Early Neolithic samples from the Tiszaszőlős-Domaháza site were treated separately for comparison.

## Discussion

Variations in the carbon and nitrogen isotope ratios suggest that there were three main dietary patterns characteristic of Great Hungarian Plain populations spanning from the Early Neolithic to the Early Iron Age (Figs 4 and 5). Additionally, variation in the δ^15^N values may be indicative of changes in agricultural and farming activities during Hungarian Prehistory.

### Mesolithic dietary pattern during the Körös Early Neolithic

The two Körös individuals from the site of Tiszaszőlős-Domaháza have lower δ^13^C and higher δ^15^N values than both the other Körös and the later period individuals. These δ^13^C values are too low for a diet based entirely on the local terrestrial resources, and suggest the incorporation of freshwater fish resources in the diet. This is supported by the faunal assemblage from the Körös levels at this site [20]. The site was dominated by wild resources including a substantial number of fish remains and mussel shells. Although fish remains are also found to some degree at other Körös sites, the isotope ratios from the other Körös individuals analysed here and in other studies [42] suggest that they did not form a substantial part of the diet elsewhere, despite their proximity to freshwater resources access. Likewise, these δ^13^C values are very similar to those found in a Mesolithic individual from a southern site (Maroslele-Pana) interpreted as freshwater resource consumer [76]. The δ^15^N values of Körös Tiszaszőlős-Domaháza individuals, nevertheless, are even higher (~1.5‰) than the Maroslele-Pana Mesolithic individual. This suggests that freshwater resources either made a greater contribution to the diet than at Maroslele-Pana, or that higher trophic level freshwater resources were consumed. Thus, the levels of fish consumption at Tiszaszőlős-Domaháza would appear to be a continuation of a Mesolithic dietary pattern, in accordance with the non-admixed Western hunter-gatherer genetic pattern found previously [13]. However, although these results are statistically significant, more samples are needed to fully understand the dietary pattern at this particular site.

### Dietary continuity from the Early Neolithic to Early Bronze Age

The remainder of the Neolithic, Copper and Early Bronze Age human isotopic data suggest a broadly similar diet based on C_3_ terrestrial resources. Nevertheless, there appears to be some temporal and regional variation. The Körös Early Neolithic samples have lower δ^13^C mean values than those of the subsequent Middle Neolithic, Copper and Early Bronze Age. Although small, the consistency of this difference suggests that it is a genuine reflection of either environmental influences on plant or animal δ^13^C values, such as increased irrigation [81], or dietary change, such as the increase in consumption of carbon enriched C_4_ plant resources (either consumed directly or by animals who ingested them). Although archaeobotanical evidence for the presence of common millet [43] and isotopic indications of C_4_ plant consumption exist in some Hungarian sites from the Early Neolithic [42], millet was probably not used as an important dietary and foddering source until later periods, as discussed below. The less negative δ^13^C values from Middle Neolithic to Early Bronze Age might indicate the inclusion of some C_4_ plant source in the diet, explaining its difference with previous Early Neolithic samples. However, the consumption was not as important as other C_3_ crops as their δ^13^C values still indicate that diet is overwhelmingly based on terrestrial C_3_ plants.

### Millet consumption from the Late Bronze Age in the Great Hungarian Plain

Although two of the Bronze Age individuals analysed in [13] have overall similar genetic affinities to modern-day Central Europeans, the isotope data presented here suggest that their subsistence was based on different resources. The isotope ratios of the Early Bronze Age individuals are similar to those observed throughout the Neolithic and Copper Age periods with a diet mostly based on terrestrial C_3_ plant resources. In contrast, the Late Bronze Age and Early Iron Age individuals have significantly higher δ^13^C‰ than the values reported for earlier periods. Marine consumption would seem extremely unlikely given the landlocked location of the GHP, but the consumption of millet is entirely possible. As mentioned above, there is some scattered evidence that broomcorn millet may have been present in Europe (including Hungary) from the Early Neolithic [40,42,43]. However, recent direct radiocarbon dating of millet grains in some Eastern and Central Europe sites has cast doubt on evidence for millet predating the Middle Bronze Age [41]. Stable isotope analysis on human bone collagen, together with the higher frequency and richness concentrations of millet grains in the archaeobotanical record, suggest that millet was consumed on a large-scale and used as an important crop in Europe from the second millennium BC. This consumption was especially noticeable from the Late Bronze Age onwards [44,45,82–84], although there is also evidence that it was present during the Middle Bronze Age in northern Italy [85]. The regular consumption of millet as a staple crop by the Late Bronze Age is thus in accordance with the archaeobotanical evidence reported in Hungary [41]. This may suggest that the two Bronze Age individuals (reported in [13]) represent separate migrations into the GHP by groups with similar genetic ancestries. Taken together, these individuals’ consumption of millet may reflect the exogenous dietary practices of migrant populations. Establishing whether millet cultivation was adopted by indigenous people through trade with people consuming it (e.g. as part of the network package from other areas, such as northern Italy [85]), or migrants introducing their crop to the local population in the GHP, requires more genetic data along with other isotope approaches (strontium and oxygen isotope analyses). The inclusion of more isotopic data from Early and Middle Bronze Age individuals will also be needed to address the question if millet was consumed as a staple crop in earlier periods, as in other areas of continental Europe [85]. Alternatively, it may also be possible that cultivation of the C_4_ crop gradually intensified from the Bronze to Iron Ages. The C_4_ plant input in the diet of Late Bronze and Early Iron Age people may originate from the direct ingestion of cultivated millet or consumption of fauna foddered with this C_4_ plant. However, without associated faunal values it is not possible to discern between these two possibilities.

### Insights into changing agricultural and farming practices

The nitrogen data exhibit variability from Early Neolithic to Early Iron Age. The results from humans (excluding the Körös Tiszaszőlős-Domaháza individuals) and domesticated animals, show significantly higher and more homogenous δ^15^N values from the Middle Neolithic onwards. Nitrogen isotopic ratios have been shown to be affected by several environmental factors (see [86] for a review). Among them, changes in the landscape due to human activities can also impact the δ^15^N values in the soil and the plants growing in it. Forest clearance resulting from slash-and-burn agriculture and animal husbandry practices have been shown to increase the nitrogen content in soils, although this pattern is not consistent in all cases [86]. A recent study on human impact on the landscape in North-East Hungary (in the Middle Tisza floodplain) [87] shows periods of woodland clearance for agricultural and farming activities in the area during Neolithic times. Especially important was the AVK Middle Neolithic period when there was an unequivocal woodland clearance and burning impact on the landscape. According to this work, this favored the spread of plant species indicative of woodland recovery following clearance. This might explain why not only the human and domesticated data from Middle Neolithic present higher δ^15^N values, so too do the wild fauna from Middle Neolithic (Figs 2B and 3B). Additionally, this study [87] suggests that clearance continued during the Early Copper Age period, which might have also increased δ^15^N values in wild fauna for the same reason. However, our data suggest that, although there is a slight increase in δ^15^N values for the wild fauna during Middle Neolithic, this difference is not statistically significant (as it is for humans and domesticates). Therefore, although the homogeneity of δ^15^N values may be explained by changes to agricultural practices, other factors may also have caused an increase of the values themselves in humans and domesticated fauna.

Previous studies in the same area show that high δ^15^N values from Late Neolithic to Copper Age might be due to both an increase of animal protein consumption and to the effect of fertilising crops with animal secondary products (e.g. manure) [25,29]. Nitrogen isotope ratios have shown to significantly increase in modern crops fertilised with animal dung in long-term experimental studies [72–74]. These results showed the manuring effect in nitrogen isotopic composition in bone collagen results, and probably the overestimation of animal protein intake in Neolithic human palaeodietary reconstructions [88]. Thus, a 9‰ to 11‰ δ^15^N range in values of human bone collagen was suggested for a combination of manured crops and animal protein intake in early Neolithic European farmers [72,73,88]. Although the sample size is small, the lack of significant variation in δ^15^N values in the wild fauna used here suggest that probably the enriched ^15^N isotopic ratios from Middle Neolithic domesticated fauna are due to the use of fodder from manured crops [86,88]. Thus, the isotopic results for human data, with values >9‰, might be indicative of both an increase of animal protein intake and consumption of agricultural products fertilised with manure from the Middle Neolithic onwards. This suggests a cultivation of manured crops earlier than previously suggested [25,29].

The decrease of the mean δ^15^N values during the Early Copper Age could be ascribed to important changes that occurred during the Neolithic and Copper Age transition. During this transition, there were crucial changes in the use of the landscape, with settlements more dispersed across the landscape and fewer nucleated sites [28]. Giblin demonstrated that these changes did not necessarily lead to abrupt changes in dietary patterns between these two periods, as originally proposed [25]. In fact, the observed decrease in δ^15^N mean values is not statistically significant, and it is the variability of individual values that increase from previous Late Neolithic ones (Fig 3B). Giblin argued that the variability during the Early Copper Age might represent more regional/local site differences in terms of protein consumption and nitrogen enrichment patterns, rather than different subsistence practices. Additional samples from more sites of north and south east Hungary will shed more light on this issue.

As well as this, Hoekman-Sites and Giblin [29] argued for an increase in animal protein consumption in the Middle and Late Copper Age, based on an increased presence of animal fat in pottery residues. The fall in δ^15^N values in our data for Late Copper Age individuals would not appear to support this interpretation. However our sample size for this time period is small (n = 3) and results must be interpreted with caution.

## Conclusions

In summary, the results of the isotopic data analysed here suggest changes in dietary patterns from Early Neolithic to Early Iron Age that are not in all cases associated with cultural and genetic shifts. The dietary practices of the individuals from the Early Neolithic Körös site of Tiszaszőlős-Domaháza is in accord with the genetic data. Their dietary isotopic profiles resemble the continuity of a Mesolithic hunter-gatherer pattern with an important reliance on freshwater resources consumption. The Mesolithic genomic signature of one of the individuals, together with similar dietary patterns, might indicate some evidence of contact between Early Neolithic communities and indigenous hunter-gatherers, as previously suggested [13]. A diet based on terrestrial C_3_ plant resources seems to be common from Early Neolithic to Copper Age periods, in accordance with the genomic stasis [13]. Although a genomic shift was shown with the advent of Bronze Age technology, isotopic results from Early Bronze Age individuals suggest that they were not accompanied by important changes in dietary pattern and that they continued with the same subsistence practices as in previous Neolithic and Copper Age times. However, the Late Bronze Age, together with Early Iron Age individuals, have significantly higher δ^13^C ratios than previous times suggesting a dietary change with a higher consumption of millet, which is in accordance with its wide cultivation in Europe from the second millennium BC. Nitrogen isotopic ratios from both domesticated fauna and humans are suggestive of consumption of fertilised crops from Middle Neolithic, which may be probable due to the use of secondary agricultural products (manure) [29].

## Supporting information

S1 Appendix Archaeological information. (DOCX)

S2 Appendix Extended methods. (DOCX)

S1 Table Stable isotope data and sample information for faunal samples analysed. All samples represent a single individual. Samples were analyzed in duplicate except for those marked with an asterisk. The δ^13^C‰ and δ^15^N‰ values represent average values of duplicate runs for each sample. (EN = Early Neolithic; MN = Middle Neolithic). Samples collected from ^1^Faunal Collection of the Archaeological Department of the Déri Museum (Debrecen, Hungary), ^2^Hungarian Natural History Museum (Budapest, Hungary).
(DOCX)

S2 Table Stable isotope data and sample information for human samples analysed. All samples were analyzed in duplicate except for those marked with an asterisk. The δ^13^C‰ and δ^15^N‰ values represent average values of duplicate runs for each sample. Samples that do not meet the quality range (C:N = 2.9–3.6) are in bold. The upper/lower M1 samples employed here correspond to a root dentine subsample belonging to the very last stage of formation of the tooth, following (AlQahtani et al. 2010) and the subsampling method in Beaumont and Montgomery, 2015. Legend: no col. = no collagen; EN = Early Neolithic; MN = Middle Neolithic; LN = Late Neolithic; ECA = Early Copper Age; MCA = Middle Copper Age; LCA = Late Copper Age; EBA = Early Bronze Age; LBA = Late Bronze Age; EIA = Early Iron Age. Infant = 0−2 years.
(DOC)

S3 Table Results of normality tests (Shapiro-Wilk W statistic) for human and faunal isotopic values. Human and faunal number of samples (N) includes the new samples reported in this study, together with the published data (references in Tables 2 and 3). *Early Neolithic samples from Tiszaszőlős-Domaháza site were treated separately for comparison In bold: *p*<0.05.
(DOCX)
