# Supplementary material for: 5000 years of dietary variations of prehistoric farmers in the Great Hungarian Plain
Source: PLoS One. 2018 May 10;13(5):e0197214. doi: 10.1371/journal.pone.0197214 (PMC5944993; doi:10.1371/journal.pone.0197214)
Supplement: S1 Appendix — (DOCX) [file pone.0197214.s001.docx]

**S1 Appendix. Archaeological information**

**Archaeological sites**

**Apc-Berekalja I** is situated at the easternmost periphery of the western Linearbandkeramik (LBK) close to the eastern Alföld Linear Pottery (ALP) cultural limit near to the Zagyva valley at the border of Pest and Heves County. It is one of the largest LBK sites in Hungary and while it was settled primarily during the late LBK and Zseliz/Želiezovce phases around 5,470-4,950 cal BC, the settlement was first occupied in the early LBK phase between 5,400 and 5,300 cal BC. Several LBK houses, pits and graves were excavated as well as other more recent features of the Late Copper Age, Early and Middle Bronze Age, Roman and Early Medieval Periods. Some of the 16 graves are from Middle and Late Neolithic periods, Copper and Bronze Age periods, as well as the Roman period.

Grave 213/6 (HUNG352) corresponds to a fragmentary juvenile skeleton laid on its right side in a strongly crouched position. It was found near a LBK house and was radiocarbon dated to the Zseliz/Želiezovce phase of the LBK. Grave 157/3 (HUNG362), from the same phase, contained a 21-22 year old female skeleton in a strongly crouched position lying on her left side, and oriented W-E. Grave 1000/12 (HUNG359) corresponds to a 53-59 year old male whose body was put on his right side into the grave pit in a slightly contracted position (ie. his legs were lifted only to the hight of the hip), and oriented W-E. It was radiocarbon dated to the earlier phase of the LBK. Grave 2 (HUNG347) was of a juvenile skeleton positioned on its right side in semi-flexed position, and the grave goods included a ceramic bowl near the lower abdomen, containing animal bones. Some *Spondylus* beads were placed around the head and waist as ornaments. These grave goods and the radiocarbon dates between ~4,530 and ~4,310 cal BC hint to the Late Neolithic Lengyel culture. Graves 686/10 (HUNG349), 714/11 (HUNG357) and 1230/14 (HUNG355) are assigned to the same age. In Grave 686/10 two skeletons were found lying close to each other in a contracted position on their right sides, and oriented NE-SW. They may have been close relatives: a 23-29 year old male (686/10.1) and a 0-5 year old child (686/10.2); the latter was not included in the analysis. Grave 714/11 (HUNG357) belonged to a 14-16 year old juvenile whose body was laid on its right side oriented W-E and to whom two pedestalled vessels were given as grave goods. Grave 1230/14 (HUNG355) consisted of a fragmentary skeleton of a 23-29 year old female. Some parts of the skeleton were missing due to ploughing. From the intact parts, a slightly crouched body could be reconstructed as lying on its right side in the grave pit, and oriented W-E. Grave 1230/15 (HUNG353) contained a skeleton of an adult female who was buried in a semi-crouched position on her right side. There were no grave goods but based on the radiocarbon dates between ~2,990 and ~2,670 cal BC, the grave can be placed in the late phase of the Copper Age Baden culture, of which there are several remains on the site. According to its radiocarbon date, Grave 928/13 (HUNG360) belongs to the Late Hatvan Culture (Early/Middle Bronze Age). The strongly contracted skeleton of a 40-59 year old male lying on its left side was excavated on the bottom of a deep pit. Samples were collected from the Archaeological Department of the István Dobó Castle Museum (Eger, Hungary).

**Berettyóújfalu-Morotva-liget** is situated in Hajdú-Bihar County, on the southern bank of Berettyó River. It contains a sequence of archaeological horizons spanning from Early Neolithic Körös to Late Medieval periods. The first excavation season in 1977 revealed three large waste pits (A, B and C), three burials (two adults from pit B and an infant from pit C), and remains of two additional individuals from disturbed graves. All features and graves included painted pottery, and were dated to the late ALP (Esztár Group) [89,90]. In 2011 an additional area of 1700 m^2^ was excavated and pits, wells, ditches and postholes of the Neolithic Körös Culture were uncovered. Obj. 44/Str. 44 (here: HUNG276) is the grave of an adult male excavated at the western border of the main area. The shallow grave pit was oval with a flat bottom. The skeleton was laid in a N–S orientation, on his left side in a flexed position with both arms folded and the hands placed in front of his face. There were no associated grave goods. The features of this burial are all typical of the funerary practices of the Körös Culture [91,92]. In the upper levels of a refuse pit the disturbed inhumation of an adult female (Obj. 47/Str. 52; here: HUNG277) was found, dating to the Körös period. The skeleton lacked a skull and was oriented in an approximately S-N direction on its right side in a strongly crouched position. Both forearms were flexed at the elbow, and with the hands positioned in front of the face. This inhumation also lacked burial goods. Samples were collected from the Archaeological Department of the Déri Museum (Debrecen, Hungary)

**Debrecen-Tócópart, Erdőalja** is situated in Hajdú-Bihar County on the northern periphery of Debrecen, eastern Hungary. Large scale rescue excavations were carried out in connection with the construction works of the M35 bypass road during 2008–2009. The site yielded materials from the Middle Neolithic ALP, Copper Age, Early Bronze Age (Makó culture), Late Iron Age (Celtic/La Tène culture), Roman imperial (Sarmatian), Late Avarian, and early and late Medieval cultural periods^93–95^. The ALP occupation is dated to the late phase of the ALP culture and includes ~50 extensive clay pits with various finds: broken pottery, fauna, stone tools and fragments of wattle and daub. Some postholes, which were structural elements of long rectangular houses, were excavated between the pits. Thirteen mostly intact graves were excavated from the ALP phase, of which eight were selected for analysis in this study.

Some of the graves were partly destroyed during the excavations works and some body parts were damaged and/or missing. Grave Obj. 223/Str. 578 (HUNG 299) was strongly destroyed and most part of the skeleton and the skull is incomplete and damaged. The individual in this grave was an adult (23-39 years old) male (?) lying on his left side in a prone contracted position with bent legs, and with his body oriented SE-NW. No observable grave goods were found. Grave Obj. 101/Str. 278 (HUNG 300) contained incomplete and damaged skeleton and cranium. The deceased juvenile was on his right side in contracted/crouched position with fully bent legs, with its body oriented E-W. No grave goods were found. Grave Obj. 221/Str. 576 (HUNG 301) contained an adult male individual lying on his left side in a contracted position with fully bent legs. The orientation of the body was SE-NW and 2 biconical limestone beads were found under the lower arms. Obj. 1412/Str.1761 (HUNG 302) grave was not well preserved (cranium and some long bones were destroyed and relatively large number of bones were missed). The grave was dug into the top of a big clay Pit 304/749. The rectangular grave pit and the axis of the body was oriented E-W. The deceased mature adult (40-59 years old) was on his left side in contracted/crouched position with fully cocked legs. His arms were also bent, but these were missed. South from the knees some painted Middle Neolithic pottery sherds came to light, which can be interpret as grave good. Some 2 meters South from this grave, another bad preserved Grave (Obj.312/Str.757) was unearthed (not included in the analysis), too (it was also dug into the same pit as the sampled Grave 1412/ 1761). Grave Obj. 930/Str. 989 (HUNG 303) contained an adult (30-35 years old) male, lying on his left side in contracted position with bent arms in front of the face. The orientation of the body was SE-NW, and without any observable grave goods. Grave Obj. 921/Str.1557 was dug into a big Middle Neolithic clay pit 920/71556. It contained a mature adult (50-59 years old) male of great stature and some bones of a child (8-10 years old) analysed here (HUNG 304), which might probably belong to another disturbed grave. Grave Obj. 1421/Str. 1770 (HUNG 306) was also disturbed during the excavation works and most of the body remains were damaged or missed. The individual belonged to a maturus (40-59 years old) female and was lying on her left side in a prone position. Her arms were probably originally bent in front of the skull. There were traces of red ochre painting observed on the cranium and on the upper part of the femur. The orientation of the body was E-W. Beneath the legs a fragmented painted pottery came to light. Obj. 994/Str. 1625 (HUNG 307) grave was strongly destroyed and containing a maturus (40-49 years old) female and an infant (? age) individual (analysed here). Only a few bones were remained, and even less stayed in situ position. No observable grave goods were found, although some Neolithic sherds remained among the bones. Samples were collected from the Archaeological Department of the Déri Museum (Debrecen, Hungary)

**Kompolt-Kígyósér** is situated between Kál and Nagyút in Heves County, eastern Hungary. In 1994, an area of 24,000 m^2^ was excavated yielding artifacts spanning from Neolithic to the Middle Ages. The ALP cultural features included a series of long and beehive-shaped pits and six burials. The ceramic finds in the pits and graves belonged to the late ALP with some Szakálhát and Bükk type pottery fragments. Grave 17/7 (HUNG372) contained no grave goods and the remains were in a fragmentary condition with many missing bones. The placement of the body on its left side in a crouched position, its radiocarbon date, and other analyses from the Debrecen laboratory, confirm its chronological attribution to the late ALP. Recent radiocarbon dates also confirm graves 24/8 (HUNG375), 25/9 (HUNG373) and 26/10 (HUNG374) attributed to the Neolithic ALP culture. The skeleton found in Grave 24/8 (HUNG375) was heavily disturbed and only some bones were found *in situ*. Despite this, the 23-29 year old female could be seen resting in a crouched position lying in her right side, and oriented NNE-WSW. The skeletal remains from grave 25/9 (HUNG373) belonged to a 44-50 year old female whose skeleton was lying in a contracted position on her left side, and oriented SE-NW. The bones were situated at a relatively shallow depth, just below the humus layer, and some body parts suffered severe damage from recent ploughing or disinterment. Grave 26/10 (HUNG374) was found in a similar condition. The skeleton of this adult female was lying in contracted position on its right side, and oriented SE-NW. Grave 5/2 (HUNG371) represented a crouched burial a headless body from a 25-34 year old male laid on its left side, and oriented SE-NW. This grave was found further away from the Neolithic grave group and, although it was not yet dated, the rituals associated with the burial are in accordance with the Neolithic features of the site. Grave 7/3 (HUNG370) was also part of the Neolithic ALP grave group of the site. It is associated with a complete skeleton laid on its left side in a highly crouched position. The grave contained a small pot typical of the ALP material of the site [96]. Despite the Neolithic archaeological context, two radiocarbon dates have dated this skeleton to the Early Bronze Age. Samples were collected from the Archaeological Department of the István Dobó Castle Museum (Eger, Hungary).

**Ludas-Varjú-Dűlő** is situated north of Ludas in the foothills of the Mátra Mountain in Heves County. This large site area was excavated between 1998–2002, revealing a rich Late Bronze Age settlement area of 18 ha [97,98]. This settlement had a monumental round structure (a ditch bordered by a palisade) which probably served as a ceremonial center on its eastern side and had an urn cemetery at its northern part. The inner part of the settlement was full of pits, but house structures were not preserved. According to the radiocarbon dates, the Late Bronze Age settlement spans the period of 1,540–1,000 cal BC. More than twenty pits yielded human burials. Pit 2161 contained two skeletons: a 2-3 year old child and an adult female (with a similar absolute date as the child, 3,130±70 uncal. BP). A subadult individual (HUNG381) was included in this study. The ceramics in the pit are typical of the Late Bronze Age Kyjatice culture which is in good agreement with the absolute radiocarbon dates. Bones of two individuals were also found in Pit 1681 belonging to an 18-22 year old female (HUNG407) and a 6-7 year old child (HUNG412). Remains of two additional individuals were excavated in Pit 1936, although only one belonging to a 47-55 year old female (HUNG 397) was used here for analysis. Pit 1935 also contained the remains of two individuals, whose remains were sparse and not lying in anatomical order. The bone remains were found in the lower layer of the pit. Only one individual (HUNG415), an 18-22 year old male, was analysed here. Human remains found in Pit 1433 (HUNG392, 23-29 year old male), Pit 2322 (HUNG389, 16-17 year old individual) and Pit 2262 (HUNG394, adult male) consisted mainly on skull bones. These were situated in varied depth, but were accompanied in each case with rich archaeological finds. A complete skeleton of a 6-8 year old child (HUNG406) was excavated in the lowest part of Pit 1851. Rich grave goods accompanied this individual in the form of unscathed vessels displayed on top of the crouched body, which was lying on its left side, and very close to the wall of the pit. Skeletons in crouched position were also found in Pit 1916 (HUNG410) and Pit 2181 (HUNG429). In the former case, the 23-29 year old female was lying on her left side, while in the latter a 35-39 year old male was laid on its back and with his legs turned slightly to the right. Pit 1058 was only 16 cm in depth and contained few, dispersed human bones (HUNG387) together with some cattle remains. The reconstruction of the depositional context revealed that these human bones had originally been deposited in a different location before being transported to Pit 1058. Additionally, Pit 1058 contained other human bones that were found out of context in ashy or charred layers indicative of some kind of cremation prior to burial. Pit 1090, with a 20 cm depth, contained burnt remains including fragments of jaw, teeth and long bones belonging to an adult male (HUNG413). The burnt human remains recovered from Pit 1751 (HUNG396) are those of a cremation burial of an infant found in a bowl, together with other intact objects including a ceramic vessel and a grinding stone, all lying in the middle layers of the pit. The 23-29 year old male skeleton in Pit 1033 (HUNG382), found lying in a crouched position on his right side, was not accompanied with grave goods and his age is uncertain. Nevertheless, based on the similarity of the burial ritual, this individual likely belongs to the Late Bronze Age.

At the southern edge of the settlement, a pre-Scythian cemetery was excavated revealing two grouping of skeletons. The first with 7 graves was very poorly preserved, while the second (with 12 graves) were better preserved. Grave 2638 (HUNG418) belonged to the second group and contained the skeleton of a 4-6 year old child [99]. The grave had been disturbed by recent ploughing. The skeleton was laid in an extended supine position. The only excavated grave good was a broken bronze pin. The other graves in the cemetery contained both ceramic and iron objects, which were used to date the cultural phase of the cemetery to that of the Early Iron Age Mezőcsát Culture. Grave 2630 belonged to a 41-45 year old female (HUNG417) who was buried on her back in an extended position, and oriented SW-NE. She was buried with a vessel well and bronze pin. Grave 2633 contained a 36-40 year old male (HUNG401) lying on his back, along a W-E direction, and covered with only a 35 cm humus layer. No grave goods were found, although it might be due to damage caused by ploughing that also destroyed the right side of the skeleton. Based on the burial ritual and body position, this individual likely belonged to the Early Iron Age Mezőcsát culture.

All samples from this site were collected from the Archaeological Department of the István Dobó Castle Museum (Eger, Hungary).

**Nagykörű-Gyümölcsös TSZ** is situated on a loess-covered elevation overlooking a former channel of the Tisza River [100]. The site was initially identified in the 1970s, when preparations for a fruit orchard brought finds typical of the “fringe” areas of the Early Neolithic Körös Culture to the surface. The extent of the surface scatter of finds suggested that the site extends over 280-300 m by 100 m, but no excavation took place until some 30 years later. In 2004 permission was granted to excavate a small trial trench, during which a skeleton was found deposited in a refuse pit. The individual (Grave 1, HUNG492) was lying in a crouched position on its right side, and was orientated NE-SW. The fill surrounding the skeleton was burnt and contained large quantities of mussel shells, which were probably consumed or deposited as part of a burial ritual. Radiocarbon dates suggest that the settlement was contemporary with that of Tiszaszőlős-Domaháza, being inhabited from 5,880-5,480 cal BC. Samples were collected from the Department of Anthropology, Hungarian Natural History Museum (Budapest, Hungary).

**Szolnok-Szanda** is situated on the northern bank of an alluvial inlet, and was first identified during a field survey in 1976. Partial excavations in the following two years uncovered an early Neolithic settlement of the Körös culture, including the burnt remains of houses along with seven burials. Based on the extent of the site it is estimated that there may originally have been a maximum of 10-12 houses at the settlement, but only 5-6 occupied concurrently [101]. Six houses were found in the excavated areas in which infilled pits were found under a number of the house structures, suggesting multiple occupation phases at the site. This is supported by the pottery typologies and radiocarbon dates from the site, which divides the site’s use into two main phases: the earliest phase with radiocarbon dates from 5,840-5,730 to 5,720-5,670 cal BC, and the later phase with radiocarbon dates from 5,720-5,670 to 5,610-5,490 cal BC. Occupation at the site may have been chronologically continuous but variable in its spatial extent, or the Körös settlement at the site may have been periodic in nature [101].

Three of the skeletons from the site were selected for isotopic analysis. The first of these, Grave 2 (SzSz 02), was the fragmentary skull and postcranial skeleton of a 6-8 year old child, who had been buried lying in a contracted position on the right-hand side in a SE-NW orientation [92]. The burial lay to the southeast of House 1, and contained no grave goods. Grave 3 (SzSz 03) contained the incomplete skeleton of a 40-46 year old gracile female found under the rubble of House 2. The skeleton had been laid on the left-hand side in a contracted position, orientated N/NE to S/SW. There were no grave goods, but objects found in the overlying house may have been part of the burial furniture and set alight as part of a burial rite [92]. Grave 4 (SzSz 04) was found in Pit 5, which lay to the southern side of House 1. The remains were the postcranial skeleton of a 51-57 year-old female, with the skull missing. The body was oriented E-W and was placed on its right-hand side in a contracted position. There were no grave goods. Samples were collected from the Eötvös Lorand University (Budapest, Hungary).

**Tiszaszőlős-Domaháza** is a small site at the northernmost settlement of the Körös culture along the Middle Tisza River. Here the Körös cultural phase includes a pit, a house plan and some graves [20]. The house plan was very regular in shape, forming an oblong of 12×5.5 m, directed NW–SE along its longer axis and consisting of a thick layer of ceramic and mussel shell fragments. In the center of its southern half were the burnt remains of a hearth. In addition to the Körös features, the site also contained remains from the earliest ALP culture, the early Bronze Age, and the Medieval period. The remains of seven Neolithic individuals were found at the site: two complete skeletons, two nearly complete skeletons identified by their dispersed body parts, and three represented by single bones. Most of the human remains were found in Pit 6. One skeleton (Grave 2-3; HUNG345), separated into two or three parts probably by later disturbance, was found in the lower (10–13^th^) layers of this pit and dated from the Körös period. Two further Körös skeletons from Pit 6 were each represented by a single element: Grave 4 (HUNG345a) by a skull, and Grave 5 (not included here) by a jaw. The two most complete skeletons (Graves 1, HUNG344; and Grave 6, HUNG346) were found in the eastern and northern sections of Pit 6, and dated to the Szakálhát group of the Late Middle Neolithic. These were standard burials in a crouched position. Samples were collected from the Archaeological Department of the István Dobó Castle Museum (Eger, Hungary).

**References**

89. Sz Máthé M. Újkőkori település Berettyószentmárton-Morotva lelőhelyen — Neusteinzeitliche Siedlung im Fundgebiet Berettyószentmárton-Morotva. Debreceni Déri Múzeum Évkönyve 1978. 1979; 35–56.

90. Szathmáry L. . Koraneolitikus (AVK) csontvázleletek Berettyószentmárton-Morotva lelőhelyről — Frühneolithische Knochenfunde aus Berettyószentmárton-Morotva, einem Fundort im Hajdú-Bihar. Debreceni Déri Múzeum Évkönyve 1978. 1979; 35–56.

91. Paluch T. The Körös culture graves. In: Makkay J, editor. The excavations of the Early Neolithic sites of the Körös culture in the Körös valley, Hungary: the final report. Volume I. The excavations: stratigraphy, structures and graves. Società per la Preistoria e Protostoria della Regione Friuli-Venezia Giulia; 2007. pp. 247–257.

92. Paluch T. Graves in the Körös culture distribution area in Hungary. In: Anders A, Siklósi Zs, editors. The first Neolithic sites in Central/South-East European transect. Volume III: The Körös culture in Eastern Hungary. Oxford: BAR International Series 2334; 2012. pp. 179–185.

93. Dani J. Preliminary report on the excavation at Debrecen-Nyulas (Toyota-Szalon), Eastern Hungary. Ősrégészeti Levelek/Prehistoric Newsl. 2007;8–9: 51–53.

94. Zoffmann Z. Human remains from the Esztár group site of Debrecen-Nyulas (Toyota Szalon), Eastern Hungary. Ősrégészeti Levelek/Prehistoric Newsl. 2007;8–9: 55–56.

95. Hadjú T, Nagy EG. Debrecen, Tócó-part, Erdőalja. Régészeti Kut. Magyarországon 2008/Archaeological Investigations Hungary 2008. 2009; 180–181.

96. Domboróczki L. Radiocarbon data from neolithic archaeological sites in Heves County (North-Eastern Hungary). Agria. 2003;39: 5–76.

97. Domboróczki L. Régészeti-kutatások Ludas, Varjú-dűlőn 1998 és 2002 között (Előzetes jelentés) — Archaeological investigations at Ludas, Varjú-dűlő between 1998 and 2002 (Preliminary Report). Régészeti Kut Magyarországon 2002/Archaeological Investigations Hungary 2002. 2004; 5–23

98. Domboróczki L. Recherches archéologiques à Ludas-Varjú-dűlő. In: Szabó M, editor. La nécropole celtique à Ludas-Varjú-dűlő. L’Harmattan; 2012. pp. 155–169.

99. Zoffmann Z. A preszkíta Mezőcsáti kultúra embertani áttekintése és újabb leletei Ludas–Varjú-dűlő lelőhelyről — Anthropological outline of the pre-scythian Mezőcsát culture and its new finds from the Ludas–Varjú-dűlő site. Anthropol Kozl. 2008;49: 35–42.

100. Raczky P. Nagykörű-Tsz Gyümölcsös (Co-operative Orchard). In: Anders A, Siklósi Zs, editors. The first Neolithic sites in Central/South‐East European transect. Volume III: The Körös culture in Eastern Hungary. Oxford: BAR International Series 2334; 2012. pp. 97–105.

101. Raczky P. Research on the settlements of the Körös Culture in the Szolnok area: Excavations at Szajol-Felsőföld and Szolnok-Szanda. In: The first Neolithic sites in Central/South‐East European transect. Volume III: The Körös culture in Eastern Hungary. Oxford: BAR International Series 2334; 2012. pp. 85–95.
