# Supplementary material for: 5000 years of dietary variations of prehistoric farmers in the Great Hungarian Plain
Source: PLoS One. 2018 May 10;13(5):e0197214. doi: 10.1371/journal.pone.0197214 (PMC5944993; doi:10.1371/journal.pone.0197214)
Supplement: S2 Appendix — (DOCX) [file pone.0197214.s002.docx]

**S2 Appendix. Extended methods.**

Collagen extraction followed a modified Longin method [77]. Bone fragments were prepared by cutting a sample of approximately 0.2-1.00 g from original bones using a handheld rotary tool with a diamond-coated cutting wheel. The outer surfaces were then abraded by using either a diamond coated burr or by means of a sandblaster. Bone samples were first weighed and then demineralised in 0.5M HCl at 4°C until soft and pliable. Demineralization time varied between bone samples, from just over a week to 3 weeks. Samples were then rinsed in deionised water three times and gelatinized in pH 3 HCl solution at 70°C for roughly 48hrs. The resulting gelatin solutions were filtered using Ezee filters and then freeze-dried. Consequently, aliquots of approximately 0.35-0.6 mg were prepared in tin capsules for mass spectrometry analyses.

Samples were analysed for the isotopic composition of carbon and nitrogen in two stable isotope specialised laboratories. The stable composition is reported as δ values per mil (‰):

$\delta=\left( \frac{R_{sample} -R_{standard}}{R_{standard}} \right)\times1000$^[102]^

where R is the isotope ratio (^13^C/^12^C, ^15^N/^14^N).

Most of the samples were analysed at the Light Stable Isotope Mass Spectrometry Laboratory of the Department of Geological Science at University of Florida, using a Thermo Electron DeltaV Advantage isotope ratio mass spectrometer coupled with a ConFlo II interface linked to a Carlo Erba NA 1500 CNS Elemental Analyzer. The accuracy and precision of the measurements, based on repeated measurements of two international laboratory standards USGS40 and USGS41, is ± 0.08‰ (1σ) for δ^13^C and ± 0.07‰ (1σ) for δ^15^N. A subset of samples were analysed at the University of Bradford Stable Light Isotope Laboratory, using a DeltaPLUS XL continuous flow isotope ratio mass spectrometer coupled via a ConFlo-III interface to a Thermo Flash EA 1112 elemental analyser. International (IAEA600, IAEACH6, CHE3, N1, and N2) and laboratory standards (a fish gel and a bovine liver) were interspersed throughout each run. Analytical accuracy based on repeated measurements of international standards is ±0.2 or better for both δ^13^C and δ^15^N. All δ^13^C results are expressed in standard delta notation relative to Vienna PeeDee Belemnite (V-PDB). All δ^15^N results are expressed in standard delta notation relative to air N_2_ (AIR).

**References**

102. Sharp Z. Principles of stable isotope geochemistry. New Jersey: Pearson Prentice Hall; 2007.
