# Supplementary material for: 5000 years of dietary variations of prehistoric farmers in the Great Hungarian Plain
Source: PLoS One. 2018 May 10;13(5):e0197214. doi: 10.1371/journal.pone.0197214 (PMC5944993; doi:10.1371/journal.pone.0197214)
Supplement: S1 Table — All samples represent a single individual. Samples were analyzed in duplicate except for those marked with an asterisk. The δ13C‰ and δ15N‰ values represent average values of duplicate runs for each sample. (EN = Early Neolithic; MN = Middle Neolithic). Samples collected from 1Faunal Collection of the Archaeological Department of the Déri Museum (Debrecen, Hungary), 2Hungarian Natural History Museum (Budapest, Hungary). (DOCX) [file pone.0197214.s003.docx]

**S1 Table. Stable isotope data and sample information for faunal samples analysed.** All samples represent a single individual. Samples were analyzed in duplicate except for those marked with an asterisk. The δ^13^C‰ and δ^15^N‰ values represent average values of duplicate runs for each sample. (EN=Early Neolithic; MN= Middle Neolithic). Samples collected from ^1^Faunal Collection of the Archaeological Department of the Déri Museum (Debrecen, Hungary), ^2^Eötvös Lorand University (Budapest, Hungary).

| **ID** | **Grave** | **Site** | **Period** | **Species** | **Element** | **δ^13^C ‰** | **δ^15^N ‰** |
| --- | --- | --- | --- | --- | --- | --- | --- |
| 0624 | Obj 16/Str 16 | Berettyóújfalu-Morotva-liget^1^ | EN | Auroch | Bone | -21.8 | 7.1 |
| 0625 | Obj 16/Str 16 | Berettyóújfalu-Morotva-liget^1^ | EN | Cattle | Bone | -19.4 | 8.9 |
| 0626 | Obj 16/Str 16 | Berettyóújfalu-Morotva-liget^1^ | EN | Donkey | Bone | -20.7 | 6.4 |
| 0627 | Obj 16/Str 16 | Berettyóújfalu-Morotva-liget^1^ | EN | Donkey | Bone | -21.0 | 7.5 |
| 0628 | Obj 16/Str 16 | Berettyóújfalu-Morotva-liget^1^ | EN | Deer | Bone | -21.0 | 6.1 |
| 0629 | Obj 16/Str 16 | Berettyóújfalu-Morotva-liget^1^ | EN | Pig | Bone * | -20.4 | 5.7 |
| 0630 | Obj 16/Str 16 | Berettyóújfalu-Morotva-liget^1^ | EN | Sheep/Goat | Bone | -20.8 | 7.0 |
| 0631 | Obj 304/Str749 | Debrecen-Tócópart, Erdőalja^1^ | MN | Pig | Bone | -20.8 | 9.5 |
| 0632 | Obj 304/Str749 | Debrecen-Tócópart, Erdőalja^1^ | MN | Cattle | Bone * | -19.8 | 9.4 |
| 0633 | Grave 7 | Szolnok-Szanda^2^ | EN | Cattle | Bone | -21.2 | 6.8 |
| 0634 | Grave 8 | Szolnok-Szanda^2^ | EN | Cattle | Bone | -20.8 | 7.1 |
| 0635 | Grave 8 | Szolnok-Szanda^2^ | EN | Cattle | Bone | -20.1 | 6.1 |
| 0636 | Grave 4 | Szolnok-Szanda^2^ | EN | Pig | Bone | -20.4 | 6.0 |
| 0637 | Grave 8 | Szolnok-Szanda^2^ | EN | Sheep/Goat | Bone | -20.1 | 6.0 |
| 0638 | Grave 8 | Szolnok-Szanda^2^ | EN | Sheep/Goat | Bone | -21.0 | 6.4 |
| 0639 | Grave 1 | Szolnok-Szanda^2^ | EN | Sheep/Goat | Bone | -20.3 | 6.3 |
| 0640 | Grave 6 | Szolnok-Szanda^2^ | EN | Sheep/Goat | Bone | -20.1 | 6.5 |
