# Supplementary material for: 5000 years of dietary variations of prehistoric farmers in the Great Hungarian Plain
Source: PLoS One. 2018 May 10;13(5):e0197214. doi: 10.1371/journal.pone.0197214 (PMC5944993; doi:10.1371/journal.pone.0197214)
Supplement: S2 Table — All samples were analyzed in duplicate except for those marked with an asterisk. The δ13C‰ and δ15N‰ values represent average values of duplicate runs for each sample. Samples that do not meet the quality range (C:N = 2.9–3.6) are in bold. The upper/lower M1 samples employed here correspond to a root dentine subsample belonging to the very last stage of formation of the tooth, following (AlQahtani et al. 2010) and the subsampling method in Beaumont and Montgomery, 2015. Legend: no col. = no collagen; EN = Early Neolithic; MN = Middle Neolithic; LN = Late Neolithic; ECA = Early Copper Age; MCA = Middle Copper Age; LCA = Late Copper Age; EBA = Early Bronze Age; LBA = Late Bronze Age; EIA = Early Iron Age. Infant = 0−2 years. (DOC) [file pone.0197214.s004.doc]

**S2 Table. Stable isotope data and sample information for human samples analysed.** All samples were analyzed in duplicate except for those marked with an asterisk. The δ^13^C‰ and δ^15^N‰ values represent average values of duplicate runs for each sample. Samples that do not meet the quality range (C:N=2.9-3.6) are in bold. The upper/lower M1 samples employed here correspond to a root dentine subsample belonging to the very last stage of formation of the tooth, following [103] and the subsampling method in [104]. Legend: no col.=no collagen; EN=Early Neolithic; MN=Middle Neolithic; LN=Late Neolithic; ECA=Early Copper Age; MCA=Middle Copper Age; LCA=Late Copper Age; EBA=Early Bronze Age; LBA=Late Bronze Age; EIA=Early Iron Age. Infant=0−2 years.

| **ID** | **Site** | **Grave Number** | **Period** | **Culture** | **Age** | **Sex (method of assignment)** | **Bone sampled** | **δ^13^C‰** | **δ^15^N‰** | **%C** | **%N** | **C:N** |
| --- | --- | --- | --- | --- | --- | --- | --- | --- | --- | --- | --- | --- |
| HUNG352* | Apc-Berekalja I | 213/6 | MN | LBK | 10-12 years | Male (DNA) | Lower M1 | -19.4 | 11.8 | 39.7 | 14.4 | 3.2 |
| HUNG359 | Apc-Berekalja I | 1000/12 | MN | LBK | 53-59 | Male | Rib | -20.0 | 10.1 | 41.8 | 14.8 | 3.3 |
| HUNG347 | Apc-Berekalja I | 2 | LN | Lengyel 3 | 13-14 | ? | Long bone | -20.4 | 10.1 | 43.3 | 15.3 | 3.3 |
| HUNG357 | Apc-Berekalja I | 714/11 | LN | Lengyel 3 | 14-16 | ? | Rib | -20.0 | 9.9 | 42.5 | 15.0 | 3.3 |
| HUNG349 | Apc-Berekalja I | 686/10.1 | LCA | Lengyel 3 | 23-29 | Male | metacarpal | -20.0 | 10.1 | 43.2 | 15.3 | 3.3 |
| HUNG353 | Apc-Berekalja I | 1230/15 | LCA | Baden | Adult | Female (DNA) | Rib | -20.5 | 10.4 | 43.6 | 15.7 | 3.2 |
| HUNG355 | Apc-Berekalja I | 1230/14 | LCA | Lengyel 3 | 23-29 | Female | Rib | -20.3 | 10.4 | 43.3 | 15.6 | 3.2 |
| HUNG360 | Apc-Berekalja I | 928/13 | EBA | Hatvan | 40-59 | Male | Long bone | -19.9 | 10.7 | 42.6 | 15.3 | 3.2 |
| **HUNG362** | **Apc-Berekalja II** | **157/3** | **MN** | **LBK** | **21-22** | **Female** | **Rib** | **-** | **-** | **-** | **-** | **no col.** |
| HUNG277 | Berettyóújfalu-Morotva-liget | 47/52 | EN | Körös | Adult | Female (morphology) | Fibula | -20.5 | 8.8 | 39.2 | 13.9 | 3.3 |
| HUNG276 | Berettyóújfalu-Morotva-liget | 44/44 | EN | Körös | Adult? | Female (DNA) | Long bone | -20.4 | 10.1 | 38.2 | 13.8 | 3.2 |
| HUNG299 | Debrecen-Tócópart, Erdőalja | 223/578 | MN | late ALP | 23-29 years | Male? | Cranial fragment | -20.0 | 10.2 | 34.9 | 12.4 | 3.3 |
| HUNG300 | Debrecen-Tócópart, Erdőalja | 101/278 | MN | late ALP | 14-16 years | ? | Long bone | -19.5 | 9.5 | 33.7 | 12.0 | 3.3 |
| HUNG301 | Debrecen-Tócópart, Erdőalja | 221/576 | MN | late ALP | 45-55 years | male | Cranial fragment | -20.4 | 10.7 | 43.4 | 15.2 | 3.3 |
| HUNG302 | Debrecen-Tócópart, Erdőalja | 1412/1761 | MN | late ALP | 40-59 years | Male (motphology)Female (DNA) | Long bone | -19.8 | 9.2 | 39.7 | 14.4 | 3.2 |
| HUNG303 | Debrecen-Tócópart, Erdőalja | 930/989 | MN | late ALP | 30-35 years | Male? (morphology) | Long bone | -20.0 | 10.4 | 40.5 | 14.7 | 3.2 |
| HUNG304 | Debrecen-Tócópart, Erdőalja | 921/1557 | MN | late ALP | 8-10 years | ? | Lower M1 | -19.7 | 10.1 | 39.1 | 14.1 | 3.2 |
| HUNG306 | Debrecen-Tócópart, Erdőalja | 1421/1770 | MN | late ALP | 40-59 years | Female | MC | -20.1 | 10.0 | 43.3 | 15.5 | 3.3 |
| HUNG307 | Debrecen-Tócópart, Erdőalja | 994/1625 | MN | late ALP | Infant? | ? | Long bone | -20.6 | 10.5 | 42.9 | 14.4 | 3.5 |
| HUNG371 | Kompolt-Kígyósér | 5/2 | MN | late ALP | 25-34 | Male | rib | -19.9 | 11.2 | 42.5 | 15.5 | 3.2 |
| HUNG372 | Kompolt-Kígyósér | 17/7 | MN | late ALP | 23-29 | Male (DNA) | Lower M1 | -19.2 | 11.4 | 39.8 | 14.6 | 3.2 |
| HUNG373 | Kompolt-Kígyósér | 25/9 | MN | late ALP | 44-50 | Female | Long bone | -20.1 | 10.9 | 42.7 | 15.2 | 3.3 |
| **HUNG374** | **Kompolt-Kígyósér** | **26/10** | **MN** | **late ALP or Tisza?** | **Adult** | **Female** | **Long bone** | **-** | **-** | **-** | **-** | **no col.** |
| HUNG375 | Kompolt-Kígyósér | 24/8 | MN | late ALP | 23-29 | Female | Long bone | -20.2 | 10.9 | 43.7 | 15.3 | 3.3 |
| HUNG370 | Kompolt-Kígyósér | 7/3 | EBA | Makó or Hatvan? | 31-40 | Female (DNA) | Lower M1 | -19.8 | 11.5 | 39.7 | 14.5 | 3.2 |
| HUNG429 | Ludas-Varjú-Dűlő | 2181 | LBA | Piliny/Kyjatice | 35-39 | Male | Rib | -18.1 | 11.5 | 43.5 | 15.3 | 3.3 |
| HUNG381 | Ludas-Varjú-Dűlő | 2161.2 | LBA | Piliny/Kyjatice | 2-3 years | Male (DNA) | Cranial fragment | -16.2 | 13.4 | 35.0 | 12.5 | 3.3 |
| HUNG382 | Ludas-Varjú-Dűlő | 1033/8 | LBA | Piliny/Kyjatice | 23-29 | Male | Rib | -17.7 | 11.2 | 42.7 | 15.2 | 3.3 |
| HUNG387 | Ludas-Varjú-Dűlő | 1058/9 | LBA | Piliny/Kyjatice | 25-34 | Male | Rib | -18.7 | 11.2 | 43.0 | 15.3 | 3.3 |
| **HUNG389** | **Ludas-Varjú-Dűlő** | **2322** | **LBA** | **Piliny/Kyjatice** | **16-17** | **?** | **Long bone** | **-** | **-** | **-** | **-** | **no col.** |
| HUNG392 | Ludas-Varjú-Dűlő | 1433 | LBA | Piliny/Kyjatice | 23-29 | Male | Cranial fragment | -17.1 | 10.2 | 43.3 | 15.2 | 3.3 |
| **HUNG396** | **Ludas-Varjú-Dűlő** | **1751** | **LBA** | **Piliny/Kyjatice** | **Infant** | **?** | **Cranial fragment** | **-** | **-** | **-** | **-** | **no col.** |
| HUNG397 | Ludas-Varjú-Dűlő | 1936/1 | LBA | Piliny/Kyjatice | 49-55 | Female | Cranial fragment | -18.1 | 11.1 | 42.9 | 14.8 | 3.4 |
| HUNG406 | Ludas-Varjú-Dűlő | 1851 | LBA | Piliny/Kyjatice | 6-8 years | ? | Rib | -19.0 | 10.8 | 42.9 | 15.1 | 3.3 |
| HUNG407 | Ludas-Varjú-Dűlő | 1681/2 | LBA | Piliny/Kyjatice | 18-22 | Female | Long bone | -17.3 | 11.6 | 43.2 | 15.7 | 3.2 |
| HUNG410 | Ludas-Varjú-Dűlő | 1916 | LBA | Piliny/Kyjatice | 23-29 | Female | Long bone | -17.4 | 10.3 | 42.2 | 14.8 | 3.3 |
| HUNG412 | Ludas-Varjú-Dűlő | 1681/1 | LBA | Piliny/Kyjatice | 6-7 years | ? | Rib | -18.6 | 11.0 | 42.7 | 14.6 | 3.4 |
| HUNG413 | Ludas-Varjú-Dűlő | 1090 | LBA | Piliny/Kyjatice | Adult | Male | Long bone | -17.6 | 11.0 | 41.4 | 14.8 | 3.3 |
| HUNG415 | Ludas-Varjú-Dűlő | 1935 | LBA | Piliny/Kyjatice | 18-22 | Male | MC | -17.4 | 10.5 | 42.3 | 15.1 | 3.3 |
| **HUNG394** | **Ludas-Varjú-Dűlő** | **2262** | **LBA** | **Piliny/Kyjatice** | **Adult** | **Male** | **?** | **-17.6** | **9.7** | **38.2** | **12.2** | **3.7** |
| HUNG401 | Ludas-Varjú-Dűlő | 2633 | EIA | Mezőcsát | 36-40 | Male | Rib | -18.2 | 10.4 | 43.2 | 15.6 | 3.2 |
| HUNG417 | Ludas-Varjú-Dűlő | 2630 | EIA | Mezőcsát | 41-45 | Female | Rib | -17.2 | 11.0 | 43.5 | 15.6 | 3.3 |
| HUNG418 | Ludas-Varjú-Dűlő | 2638 | EIA | Mezőcsát | 4-6 years | Male (DNA) | Rib | -14.4 | 10.8 | 40.0 | 14.5 | 3.2 |
| HUNG492 | Nagykörü-Gyümölcsös TSZ | 1 | EN | Körös | 50-55 years | female | Femur | -20.5 | 10.5 | 30.1 | 11.0 | 3.2 |
| SzSz 02 | Szolnok-Szanda | 2 | EN | Körös | 6-8 years | ? | Cranial fragment | -20.2 | 10.4 | 40.0 | 14.2 | 3.3 |
| SzSz 03* | Szolnok-Szanda | 3 | EN | Körös | 40-46 years | Female (morphology) | Long bone | -20.8 | 11.6 | 38.9 | 13.5 | 3.4 |
| SzSz 04 | Szolnok-Szanda | 4 | EN | Körös | 51-57 years | Female (morphology) | Lower M1 | -20.6 | 11.0 | 40.6 | 14.4 | 3.3 |
| HUNG345 | Tiszaszőlős-Domaháza | 2-3 | EN | Körös | 31-37 years | Male (morphology) | Lower M1 | -22.5 | 13.1 | 38.6 | 13.8 | 3.3 |
| HUNG345a | Tiszaszőlős-Domaháza | 4 | EN | Körös | 15-17 years | Male (DNA) Female (morphology) | Cranial fragment | -22.6 | 12.9 | 39.3 | 14.3 | 3.2 |
| HUNG344 | Tiszaszőlős-Domaháza | 1 | MN | late ALP | 40-46 years | Male (morphology) | Upper M1 | -20.5 | 12.2 | 39.2 | 14.2 | 3.2 |
| HUNG346 | Tiszaszőlős-Domaháza | 6 | MN | late ALP | 34-40 years | Male | Lower M1 | -20.0 | 9.9 | 39.5 | 14.1 | 3.3 |

103. AlQahtani SJ, Hector MP, Liversidge HM. Brief communication: The London atlas of human tooth development and eruption. Am J Phys Anthropol. 2010; 142(3):481–90.

104. Beaumont J, Montgomery J. Oral histories: a simple method of assigning chronological age to isotopic values from human dentine collagen. Ann Hum Biol. 2015; 4460:1–8.
