# Supplementary material for: 5000 years of dietary variations of prehistoric farmers in the Great Hungarian Plain
Source: PLoS One. 2018 May 10;13(5):e0197214. doi: 10.1371/journal.pone.0197214 (PMC5944993; doi:10.1371/journal.pone.0197214)
Supplement: S3 Table — Human and faunal number of samples (N) includes the new samples reported in this study, together with the published data (references in Tables 2 and 3). *Early Neolithic samples from Tiszaszőlős-Domaháza site were treated separately for comparison In bold: p<0.05. (DOCX) [file pone.0197214.s005.docx]

**S3 Table. Results of normality tests (Shapiro-Wilk W statistic) for human and faunal isotopic values.** Human and faunal number of samples (N) includes the new samples reported in this study, together with the published data (references in Tables 2 and 3). *Early Neolithic samples from Tiszaszőlős-Domaháza site were treated separately for comparison. In bold: *p*<0.05.

| **Samples** | | **N** | **δ^13^C ‰** | | **δ^15^N ‰** | |
| --- | --- | --- | --- | --- | --- | --- |
|  |  |  | **Shapiro-Wilk W** | ***p-*value** | **Shapiro-Wilk W** | ***p-*value** |
| Human samples by period | E.Neolithic* | 2 | 1.000 | 1.000 | 1.000 | 1.000 |
|  | E. Neolithic | 14 | 0.969 | 0.862 | 0.951 | 0.569 |
|  | M. Neolithic | 71 | 0.985 | 0.573 | 0.982 | 0.404 |
|  | L. Neolithic | 47 | 0.977 | 0.463 | 0.968 | 0.226 |
|  | E. Copper Age | 26 | 0.847 | **0.001** | 0.959 | 0.367 |
|  | M. Copper Age | 20 | 0.962 | 0.576 | 0.943 | 0.267 |
|  | L. Copper Age | 3 | 0.987 | 0.780 | 0.750 | **<0.001** |
|  | E. Bronze Age | 2 | 1.000 | 1.000 | 1.000 | 1.000 |
|  | L. Bronze Age | 11 | 0.924 | 0.356 | 0.945 | 0.579 |
|  | E. Iron Age | 3 | 0.930 | 0.490 | 0.964 | 0.637 |
| Domesticated animals by period | E. Neolithic | 42 | 0.975 | 0.488 | 0.927 | **0.011** |
|  | M. Neolithic | 60 | 0.982 | 0.534 | 0.962 | 0.058 |
|  | Copper Age | 20 | 0.942 | 0.266 | 0.827 | **0.002** |
| Wild animals by period | E. Neolithic | 6 | 0.874 | 0.242 | 0.981 | 0.957 |
|  | M. Neolithic | 5 | 0.754 | **0.033** | 0.845 | 0.179 |
|  | Copper Age | 6 | 0.959 | 0.815 | 0.972 | 0.908 |
